# Integrated remote sensing and field-based approach to assess the temporal evolution and future projection of meanders: A case study on River Manu in North-Eastern India

**DOI:** 10.1371/journal.pone.0271190

**Published:** 2022-07-20

**Authors:** Jatan Debnath, Gowhar Meraj, Nibedita Das Pan, Kesar Chand, Sagar Debbarma, Dhrubajyoti Sahariah, Carlo Gualtieri, Shruti Kanga, Suraj Kumar Singh, Majid Farooq, Netrananda Sahu, Pankaj Kumar

**Affiliations:** 1 Department of Geography, Gauhati University, Guwahati, Assam, India; 2 Department of Ecology, Environment and Remote Sensing, Government of Jammu and Kashmir, Kashmir, India; 3 Centre for Climate Change & Water Research, Suresh Gyan Vihar University, Jaipur, Rajasthan, India; 4 Department of Geography & Disaster Management, Tripura University, Suryamaninagar, Tripura, India; 5 G.B Pant National Institute of Himalayan Environment, Himachal Regional Centre, Mohal-Kullu, Himachal Pradesh, India; 6 Department of Structures for Engineering and Architecture, University of Naples Federico, Naples, Italy; 7 Centre for Sustainable Development, Suresh Gyan Vihar University, Jaipur, Rajasthan, India; 8 Department of Geography, Delhi School of Economics, University of Delhi, Delhi, India; 9 Institute for Global Environmental Strategies, Hayama, Kanagawa, Japan; Đại Học Duy Tân: Dai Hoc Duy Tan, VIET NAM

## Abstract

A common phenomenon associated with alluvial rivers is their meander evolution, eventually forming cutoffs. Point bar deposits and ox-bow lakes are the products of lateral bend migration and meander cutoff. The present study focuses on identifying the meanders of River Manu and their cutoffs. Moreover, this study compares the temporal evolution and predicts the progress of selected meanders of River Manu. In the present research, the Survey of India topographical map, satellite imagery, and geographic information system (GIS) technique were used to examine the evolution of the Manu River meander. Subsequently, a field visit was done to the selected cutoffs and meanders of River Manu to ascertain the present status and collect data. It has been observed that many cutoffs have undergone temporal changes, and their sizes have decreased. Some have become dried or converted to agricultural fields. The width of River Manu has decreased in all the selected bends from 1932 to 2017. The sinuosity index has changed from 2.04 (1932) to 1.90 (2017), and the length of the river has decreased by 7 km in 85 years (1932–2017). The decrease in length is evident from lowering the number of meanders. Uniformity coefficient and coefficient of curvature of the bank soil samples were calculated, indicating that the soil is poorly graded and falls under the cohesionless category. Based on cross-section analysis, sediment discharge, grain-size analysis of the bank material, channel planform change, and radius of curvature, it can be stated that almost all the selected bends have the probability of future cutoff. The highest probabilities were observed in bend 3 (Jalai) and bend 4 (Chhontail). This work is aimed to provide planners with decisions regarding the construction of roads and bridges in areas that show the huge dynamicity of river meandering.

## Introduction

Meander is a common channel form observed in both sedimentary and non-sedimentary environments [1]. Meander is a highly nonlinear dynamic system that produces complex and fascinating planimetric patterns [2]. A river of any volume may assume a meandering course, alternately eroding sediments from the outside of a bend and depositing them on the inside [3]. Research related to the morphology and evolution of meandering rivers has attracted the attention of the scientific community, especially in the fields of fluvial geomorphology [4], fluid mechanics [5, 6], and hydraulic engineering. According to Leopold and Wolman, 1960 [7] and Dietrich and Smith, 1983 [8], point bar deposits and meander curvatures in meandering rivers strongly affect flow velocity and accelerate channel migration owing to bank erosion. The progressive migration of a meander due to bank erosion and point bar growth produces cross-sections of scroll-shaped ridges and swales subsequent to the curve of the channel [9]. The meandering channels usually undergo significant transformation over time, which eventually produces interesting planimetric patterns and features such as cutoffs (ox-bow lakes) [10, 11]. The collapse of a concave bank and a point bar deposition along the convex bank pushes and pulls the entire bend to migrate laterally until a cutoff threshold is reached [12, 13]. Meander cutoff generally includes the neck and chute cutoff. If a cutoff takes place to avoid the self-intersection of two reaches that come in contact, it is called a neck cutoff; otherwise, it is known as a chute.

**Fig 1:**
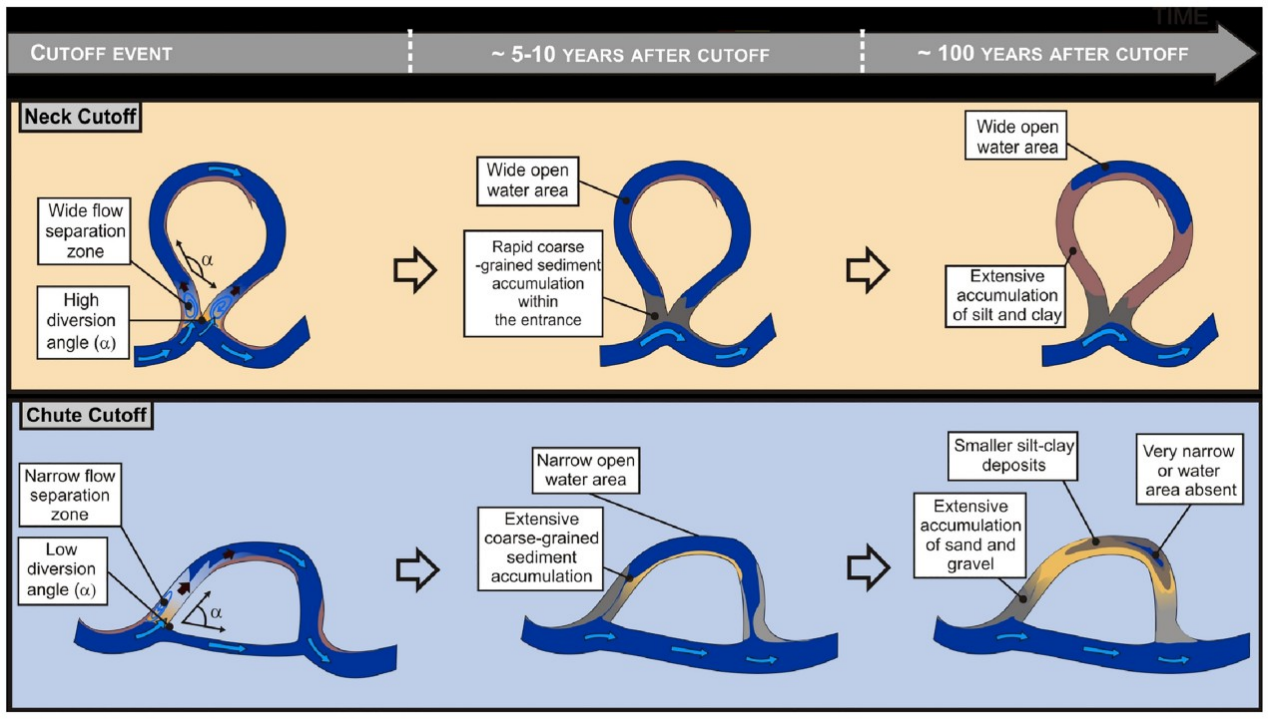
Schematic representation of the geomorphic evolution of meander cutoff [14].

Cutoffs alter the river geometry by eliminating the meanders [14], which reduce sinuosity, reshape the bend [15] and maintain its amplitude [16] (Fig 1). Apart from stimulating the landscape evolution, this complex transformation is also associated with the economic and social consequences of bank erosion. The dynamic function of a meander cutoff lies in the fact that the river has to erode a significant portion of land in order to attain a straight course. Therefore, large volumes of sediments are transferred, which can be equivalent to those resulting from 60 years of erosion by channel migration [17]. According to Dieras, 2013 [14], sediments eroded by channel incisions are redeposited in large volumes on the bars directly downstream of the cutoff, which probably accelerates the channel migration opposite to these bars. Therefore, in this entire cutoff dynamic process, bank erosion and sediment deposition play significant roles. Moreover, the meander is a geomorphic feature of a river in which maximum erosion is observed. On the other hand, a cutoff can also take place suddenly after a high-magnitude flood event. The cutoff period is much shorter (typically happens in days or weeks) when compared with the prolonged evolution time of meanders [13, 18]. Although numerous studies have been performed to understand the formation, the process of cutoff and the channel adjustment after the cutoff using flume experiments and field observation [6, 13, 17–23], the prediction of the future evolution of a meander is still uncertain. Previous studies mainly used satellite imageries, bank erosion models, and artificial rivers to understand the process of cutoff and prediction of the future evolution of meander. There is a need to integrate remote sensing/ GIS techniques and field-generated data. It will help understand the process and evolution of cutoff of a particular meander bends along with its direction of lateral migration, channel morphology, the status of the various hydrological characteristics, and bank stability.

In the scientific field of the fluvial river system, several numerical models like Support Vector, ARIMA, and Artificial Neural Network [24, 25] are applied to predict channel migration and meander evolution. According to Guo et al., 2019 [26], information concerning the dynamics of river meandering is embedded in their planforms. Asahi et al., 2013 [27] suggested that the interrelationship of several natural phenomena produces the shape of natural river meanders. When the relevant field-based observational input parameters are determined, it is possible to understand the meandering process under current conditions to determine their future evolution. The present study rigorously analyzed hydrological variables, bank strength, angle, bed topography, and direction of channel migration to understand the current conditions of the selected meanders of the River Manu North-East India for proposing their probable future paths.

River Manu in NE India is famous for its dynamic and winding nature, and a large number of people are displaced and lose their property due to it [28]. Deb and Ferreira, 2014 [24] have stated that the riverbanks are experiencing a moderate to high erosion rate in the Manu River basin, which has posed hazards to the surrounding bank line areas. Over 85 years (1932–2017), dynamic changes have been noticed in River Manu. There is an urgent need to understand the spatiotemporal meandering changes in this river for assessing the future location of meanders in the river. This is particularly important for planning structural measures such as bridges. Since the lateral migration rate or erosion capacity can impact the bridges, narrowing down on locations that can be future meanders is vital for such developmental activities. Moreover, such studies are also used to determine perfect areas for constructing a new road away from the migrating meandering channel. Although riverbank erosion depends on bank stability, channel shape (especially meander) influences it extensively and is therefore critical to analyze. The present study aims to identify the critically dynamic meanders of River Manu and their cutoff and assessment of bank erosion, compare the temporal evolution of the selected meanders of this river, and predict the future progress of these river meanders. Assessment of paleochannels using an integrated field and geoinformatics based approach opens a research domain through which the shifting trend, temporal evolution, and future projections of the meanders can be more efficiently examined than modeling-only approaches. The field-generated data regarding channel topography, flow velocity, discharge, width, wetted perimeter, water level, bank soil properties, and other parameters provide an actual scenario of a river meander. The methods and the results of this work shall be beneficial for the engineers and planners formulate the necessary action plans. Hence, it will offer an opportunity to understand the river channel modifications and their implications regarding the displacement of people and the loss of agricultural land and infrastructures near the rivers. A deep understanding of fluvial processes and predicting future evolution can be useful in river engineering, land use planning, and river restoration [29].

## Study area

**Fig 2:**
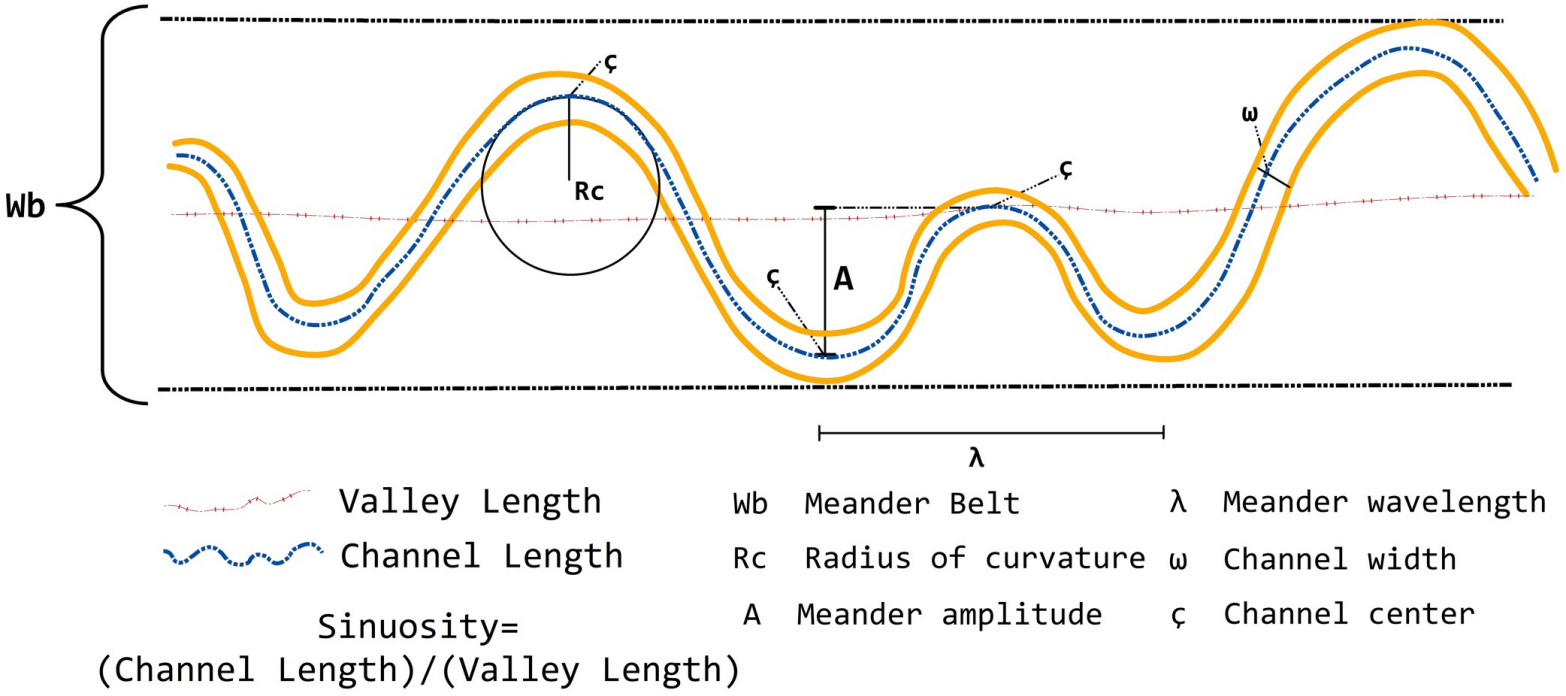
Schematic representation of meander geometry [30].

Meander planform can be defined quantitatively by meander sinuosity index (SI), meander wavelengths, meander amplitude, a radius of curvature, and channel width (Fig 2). SI deals with the meandering nature of the river and is the ratio between the current length and the straight length of the river. Straight and meandering channels are described by sinuosity, which is the ratio of the channel length to the valley length or the ratio of the valley slope or the channel gradient, as measured over the same valley length (Table 1).

**Table 1:** Classification of river channels.

Type	Morphology	Sinuosity
Meandering	Straight	>1.5
Braided	Two or more channels with bars and small island	<1.3
Straight	Single channel with pools and riffles, meandering thalweg	<1.5
Anastomosing	Two or more channels with large, stable islands	>2.0

Meander wavelengths are the linear and curvilinear lengths between the apexes or the first inflection points of successive bends on the same side of the river, respectively [30]. Meander amplitude is the lateral distance between tangential lines drawn to the center channel of two successive meander bends [7]. The size of the meander bends is analyzed by measuring the radius of curvature, which is estimated by drawing a best-fit arc through the meander bend (Fig 2). The loop radius is considered to be the straight line perpendicular to the down-valley axis intersecting the sinuous axis at the apex.

The planform evolution of meandering rivers occurs due to mutual adjustments between meandering forms and processes. The interactions among the river flow, sediment transport, and channel bed morphology determine the spatial and temporal patterns of the sediment erosion, transportation, and deposition and thus the evolution of the meandering river [7, 30].

**Fig 3:**
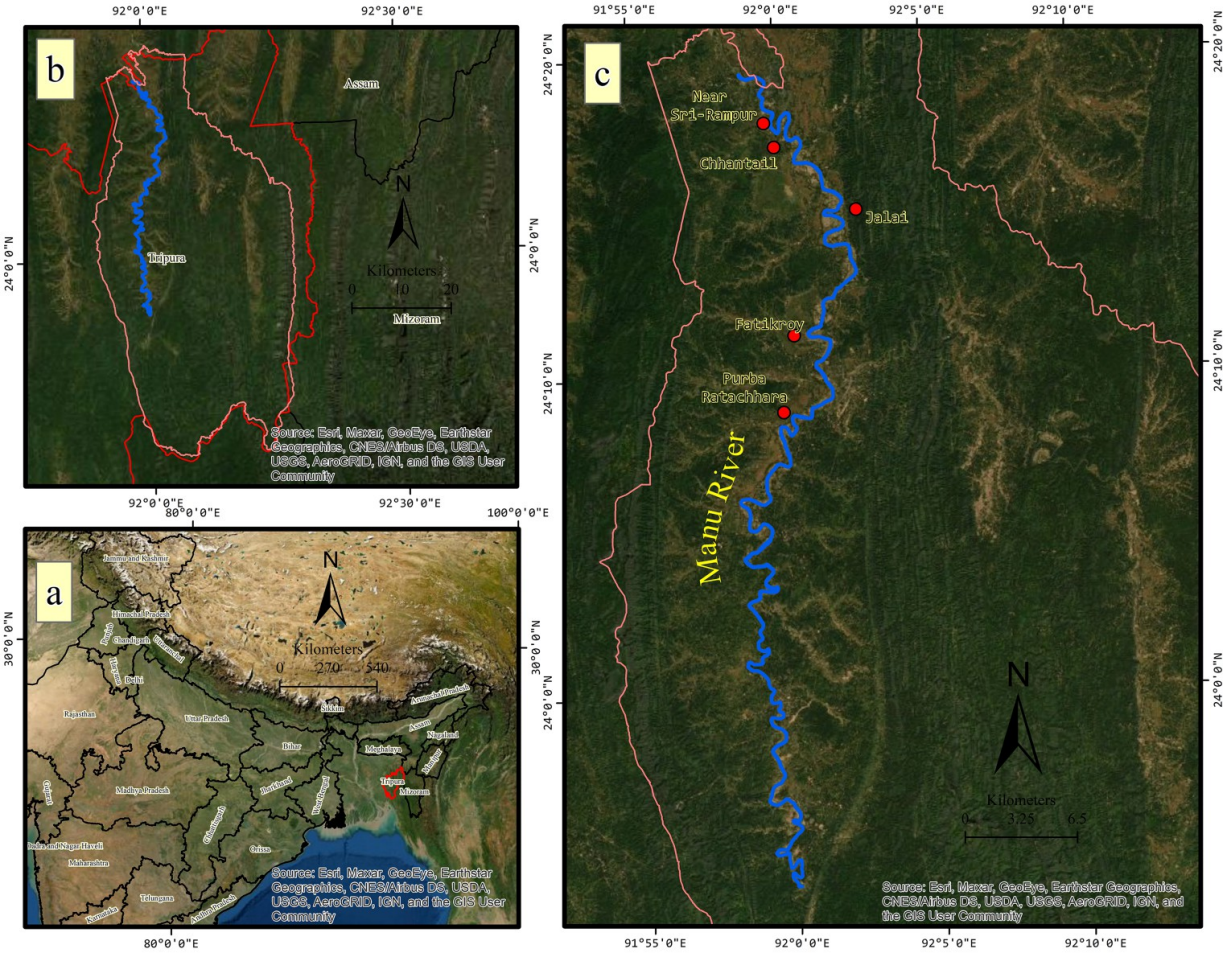
Location map of the study area, (a) The location of the state of Tripura with respect to the Union of India, (b) The basin boundary of the river Manu (pink), (c) River Manu. The map coordinates are in UTM WGS 84 North.

The Manu River rises from the Kohosib peak of the Sakhantlang hill range in the state of Tripura, the North-Eastern region of India (Fig 3). Manu is the second-longest river in the state, with 138 km. It has a catchment area of 1979 km^2^, covering 18.86 percent of the state’s total geographical area. The river has an annual flow of about 1,700,34,000 m^3^, which is 21.44 percent of the total flow. The research involves the analysis of the paleochannels, particularly cutoffs, and the evolution of selected meanders of Manu River over 89 km, extending from 23°53′58.83″N latitude and 92°00′01.73″E longitude to 24°19′30.06″N latitude and 91°58′50.41″E longitude, i.e., from the vicinity of Durgacherra to Chandipur. Five bends of the river were selected for the analysis of temporal evolution, namely, bend 1 (Purba Ratachhara), bend 2 (Fatikroy), bend 3 (Jalai), and bend 4 (Chhontail), and bend 5 (near Srirampur). This area corresponds to the lower plain of the river, where several meanders and cutoffs were found.

## Materials and methods

In the present research, the Survey of India (SoI) topographical map (1932), satellite imagery (Landsat MSS 1975, Landsat TM 1995, and Landsat OLI 2017), were used to identify the evolution of the Manu River meanders at selected segments using geospatial analysis [31, 32]. The Landsat imageries, available on the USGS website free of cost, provided valuable information for the identification and monitoring of natural as well as anthropogenic environments [33–38]. Remote sensing (RS) and GIS techniques are the most effective tools for quantifying channel alterations on a spatiotemporal scale. The obtained Landsat datasets were pre-geo-referenced using UTM zone 46N projection and WGS 84 datum. At the same time, the SoI topographical map (1932) was geo-referenced using a similar projection and datum in ArcGIS [39–41]. Detailed specifications of the datasets are listed in Table 2. The basin area was delineated over the base map, i.e., the topographical map of 1932.

**Table 2:** Basic information of satellite imageries used for the study.

Data sets	Platform	Path/Row	Resolution/Scale	Year	Source
Topographical Map	-	-	1:63360	1932–1933	Survey of India
Landsat MSS	Landsat 1	136/044	60 m	1975	USGS
Landsat TM	Landsat 5	136/044	30 m	1995	USGS
Landsat OLI	Landsat 8	136/044	30 m	2017	USGS

### Meander evolution and analysis

**Fig 4:**
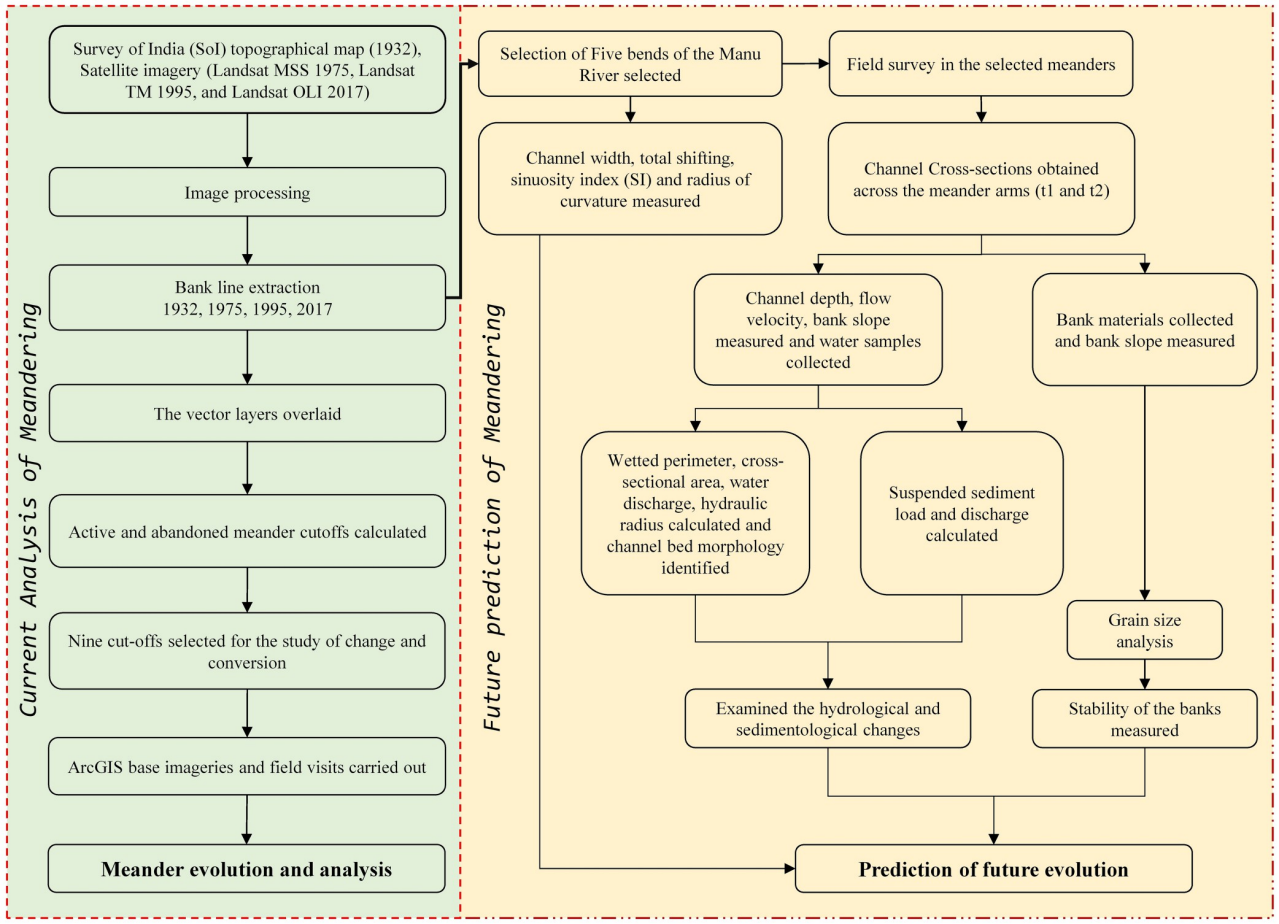
Conceptual framework of the methods used.

Topographical map 1932 was used as the base year, and Landsat data for 1975, 1995, and 2017. Four equal segments were marked to identify the meander evolution of the river from Nalkata Barrage to Chandipur (near the Bangladesh border). Since the lower course of the river experiences more erosion than the upper course, only this part was selected for the study of meander evolution. The total meander cutoff was calculated for all the study years, i.e., 1932, 1975, 1995, and 2017. The riverbank line was recognized from the SoI topographical map (1932) and satellite images (1975, 1995, and 2017). The demarcated bank lines were digitized for both the right and left banks using ArcGIS 10.1. Subsequently, the polygon vector layers were overlaid, and the overall active and abandoned meander cutoffs of the channels within these four segments were calculated. Among the total meander cutoffs, nine were selected for the study related to the changes and conversions. The present condition of the cutoff was also identified using these nine meander cutoffs. ArcGIS online base imageries were examined to understand the conversion and present status of the selected cutoffs. Subsequently, field visits were carried out to the selected cutoffs to ascertain their present status (Fig 4).

### Prediction of the future evolution of the meander

In this study, the approach to predict the future evolution of a meander based upon the combined use of remote sensing and field data which was applied to five bends of the Manu River. Bend 1, Purba Ratachhara; Bend 2, Fatikroy; Bend 3, Jalai; Bend 4, Chhontail; and Bend 5, Srirampur (Fig 3). These bends were selected based on the acuteness of the meander. For identifying the potential areas of new cutoffs and the future migration of meanders, evolution of meander channel width, total shifting, sinuosity index (SI), and radius of curvature were measured. Moreover, cross-sections, grain size distribution, and suspended sediment discharges were calculated for the selected meander bends. The arms of the meander bend, i.e., t1 (for upstream) and t2 (for downstream), were marked, and the field visits and sample collections were done at these points.

The river’s width is defined as the distance between the two banks. The temporal analysis of width to identify banks erosion and deposition was almost identical to as reported by Chakraborty and Mukhopadhay, 2015 [42]. The present study examined the width and migration of River Manu for the studied years in the selected five bends using the geospatial analysis. The riverbank line was identified and delineated from the selected toposheet and satellite images. The recognized bank lines for the left and right banks of the river were digitized using ArcGIS 10.1 software. Subsequently, these polygon vector lines were overlaid, and the overall area under migration of the channel was quantified from this overlaid map. Arm-wise (t1 and t2) channel widening, temporal shifting or migration, and the direction of shifting for each location were measured from the five selected meander bends of the river course for the periods of 1932–33 (SoI topographical map), 1975, 1995, and 2017 (satellite imagery). The point bars and active corridors were also taken into consideration.

The SIs of the river was calculated for the years 1932, 1975, 1995, and 2017. The following formula for SI was proposed by Schumm, 1963 [43],

$$Channel\;sinuosity=\;\frac{\text{OL}}{\text{EL}}$$ (1)

Where OL is the observed (actual) channel length, and EL is the expected straight path of the channel. Brice, 1964 [44] opined that a channel could be classified into three types based on SI, i.e., straight (SI < 1.05), sinuous (SI 1.05–1.5), and meandering (SI > 1.5), whereas Selby, 1985 [45] classified the channels as presented in Table 3.

**Table 3:** Sinuosity index of River Manu.

Year	Actual length (km)	Straight length (km)	Sinuosity index	Channel pattern
1932	97.12	47.57	2.04	Meandering
1975	96.61	47.44	2.04	Meandering
1995	88.77	47.43	1.87	Meandering
2017	89.88	47.40	1.90	Meandering

A field survey was conducted across the river to examine the hydrological and sedimentological changes. The cross-sections were considered across the arms (t1 and t2) of the selected meander bends. The requisite data on channel depth, flow velocity, bank slope, SSC, and grain size were generated using different instruments. Channel cross-sections were obtained using *Dumpy Level (BP-DL007 model)* and a measuring tape (100 m). The bank slopes were gauged using a *Clinometer Compass of 75 mm*. Flow velocity was measured with the help of a *Pigmy type current meter*.

Moreover, water samples were collected in 1000-mL plastic bottles to assess the SSC using a 125-mm filter paper (*Whatman*) and weigh balance (*KERN EMB 200–2*). The grain size of the bed sediment and bank material samples was analyzed using Sieve Shaker with Analytical Test Sieve (ASTM). *South Auto Level* instrument with ±0.30ʺ accuracy recorded significant rises and falls in the riverbed. Staff readings along the traverse across River Manu at 2-m intervals were recorded upstream (t1) and downstream (t2) of the selected meander bends to identify the channel depth.

Clinometer Compass was used to measure the bank slope [46]. The bank height at different points was recorded utilizing the survey staff, and the degree of the slope was directly read from the compass. The values were plotted to obtain the valley profile of the river at the selected sites.

The flow velocity of the river was determined by counting the number of revolutions of the bucket wheel of the Pigmy Water Current Meter over 10 seconds. The velocity was measured in the upper, middle, and lower layers of the river at 2-m intervals along the width. The mean velocity of the three layers at each point was calculated; finally, by averaging all the mean values, the flow velocity of the channel was computed in meter/second (m/s).

Water discharge at a particular section was estimated using the following formula:

Q=A×V (2)

Q is the water discharge, A is the cross-sectional area, and V is the flow velocity.

Grain size and suspended sediment load were estimated to comprehend the sedimentological alterations. Water samples collected immediately upstream and downstream of the confluence were tested in the laboratory to identify the river’s changes in suspended sediment concentration (SSC) after confluence.

To determine the SSC, water samples (1 L) were filtered through a 125-mm filter paper, and the following formula proposed by Old et al., 2005 was applied [47].


$$\text{SSC}\;\left(\text{mg}/\text{L}\right)=\frac{\text{Dry}\;\text{weight}\;\text{of}\;\text{suspended}\;\text{sediment}\;\left(\text{mg}\right)}{\text{Volume}\;\text{of}\;\text{water}\;\text{sample}\;\left(\text{L}\right)}$$ (3)

On the other hand, suspended-sediment discharge, which is generally expressed in millions of tons per day, was evaluated using the following empirical formula given by Gray, 2008 [48],

Qs=Qw×Cs×k (4)

Where Qs = Suspended sediment discharge in metric tonnes per day (mt/day); Qw = Water discharge in cubic meters per second (m^3^/second); Cs = Mean concentration of the suspended sediment in the cross-section in milligrams per liter (mg/l); K = coefficient based on the unit of measurement of the water discharge, which is 0.0864 when the discharge is expressed in cubic meters/second (m^3^/second).

The grain size of the collected bedload samples was analyzed using the sieving technique. In this method, the grain size’s median value (ϕ50) was considered. To determine whether the soil is coarse or fine, the percentage of material passing through each sieve was calculated. According to ISSCS (Indian Standard Soil Classification System) and USCS (Unified Soil Classification System), fine-grained soils are those in which >50% of the material has a particle size of <0.075 mm, i.e., more than 50% of the material should pass through sieve #200 (0.075 mm) [49]. The well-graded or poorly graded nature of the soil can be known by determining the C_u_ (coefficient of uniformity) and C_C_ (coefficient of curvature) values.

$$\text{Cu}={\text{D}}_{60}/{\text{D}}_{10}$$ (5)

$$\text{Cc}={\left({\text{D}}_{30}\right)}^{2}/\left({\text{D}}_{60}0\times {\text{D}}_{10}\right)$$ (6)

Where, D_60_ = Diameter corresponding to 60% fine in the grain-size distribution; D_30_ = Diameter corresponding to 30% fine in the grain-size distribution; D_10_ = Diameter corresponding to 10% fine in the grain-size distribution.

## Results and discussion

### Meander evolution and analysis

#### Sinuosity index

The SIs of the river was calculated for the years 1932, 1975, 1995, and 2017. The river exhibited a meandering pattern in all the years mentioned above, although its sinuosity magnitude changed temporally. In 1932 and 1975, the river had an SI of 2.04 and decreased to 1.87 in 1995 due to the straightening of the channel. Similarly, the meandering pattern of the river was observed in 2017, with an SI of 1.90. Thus, in 1975 and earlier, the river was much more sinuous in nature, which had been decreased substantially in 2017, with a higher number of abandoned channel remnants (cutoffs). However, it should be noted that there were more cutoffs during 1932, even though the river had a higher SI. This observation could be explained by the reasoning that those were earlier cutoffs formed before 1932 when the river was straightening, and as the years passed, the cutoffs dried up or got sedimented.

#### Channel length over the years

The actual length of the channel showed a significant decrease. The highest length of 97.12 km was observed in 1932, which decreased to 96.61 km in 1975 and 88.77 km in 1995. However, in 2017, the length increased slightly to 89.88 km. The number of meander bends decreased from 111 in 1932 to 78 in 1975, 71 in 1995, and 68 in 2017. Therefore, the channel length decreased due to the straightening of the river course.

#### Investigating the cutoffs

**Fig 5:**
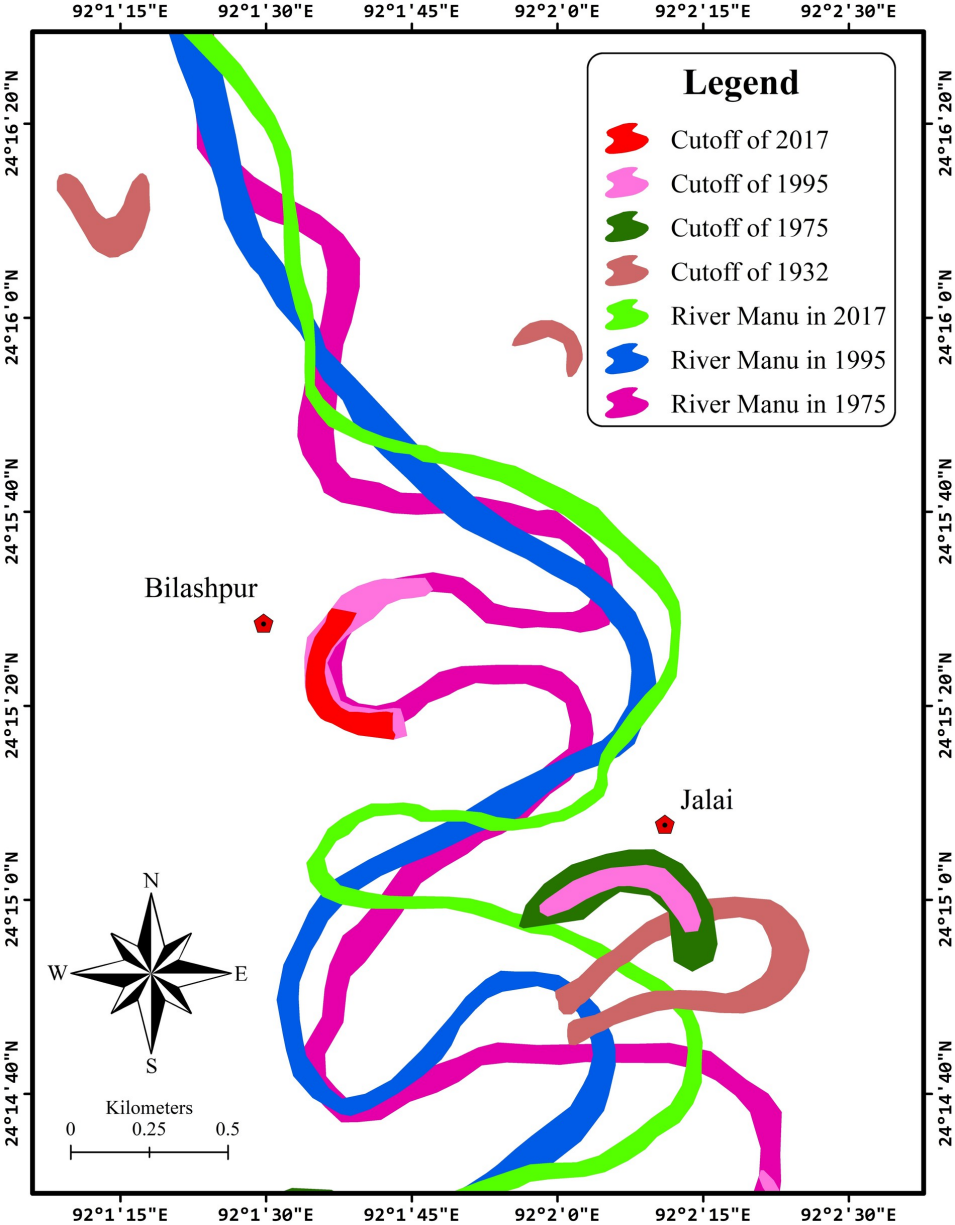
Neck cutoff of meander in the River Manu (at Segment 3).

Cutoffs are remnants of the paleochannels, which can trace the earlier path of the river and its migration (Fig 5). A cutoff is a significant element of the river meander family, which is important for studying the evolution of river meanders. For ease of understanding, the studied river was segmented into four unequal parts to demonstrate the cutoffs for different years, as follows: Segment 1, from Durgacherra to Paschim Karamchhara; Segment 2, from Paschim Karamchhara to Sonaimuri; Segment 3, from Sonaimuri to Kaulikura; Segment 4, from Kaulikura to Chandipur (border).

#### Occurrences and developments in cutoffs

**Fig 6:**
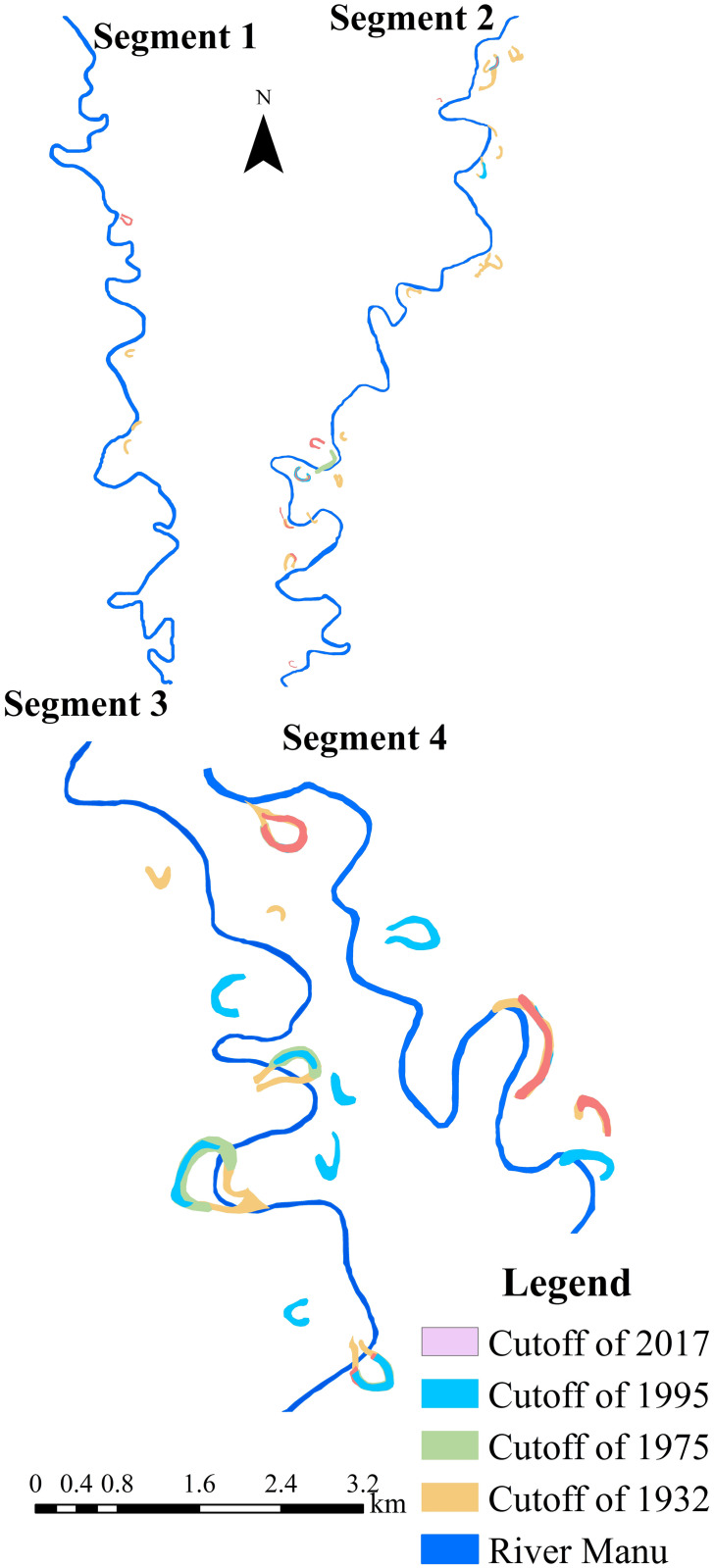
Map showing overlaid cutoffs of different years.

River Manu exhibit frequent channel shifts from left to right and vice-versa. It leads to aggradations and degradations in the riverbank because of its broad characteristics of lateral erosion. The selected parts of the river course for the periods of 1932 (SoI topographical map), 1975, 1995, and 2017 (satellite imagery) were overlaid using ArcMap 10.1 to observe the developments in the cutoff of the river. From Fig 6, it could be perceived that the meander cutoffs were changed remarkably over the years.

The cutoffs decreased from 24 in 1932 to only 5 in 1975. The number of cutoffs again demonstrated a positive trend between 1995 and 2017 (Table 4). As the meandering river straightens, it abandons the long route (meander) and forms cutoffs. The increase in cutoffs could be attributed to the fact that the river is straightening its path.

**Table 4:** Number of cutoffs observed in the selected years of study.

Year	Number of cutoffs				Total
	Segment 1	Segment 2	Segment 3	Segment 4	
1932	3	13	5	3	24
1975	Nil	1	3	1	5
1995	Nil	4	6	5	15
2017	1	7	4	3	15

A temporal decrease in cutoffs may be due to sediment deposition, drying up of the cutoff, or conversion to agricultural land. It should be noted that only cutoffs with water had been depicted on the map, and the numbers indicate the cutoffs that had displayed drastic changes over the years.

#### Conversion of cutoffs

Cutoffs undergo drastic changes temporally. Alterations such as decreased area, drying up or becoming barren, and conversion to agricultural land can be observed. Channel fillings are deposits made in abandoned cutoff channels. Significant changes in the area after applying the overlay of various years are shown in Table 5.

**Table 5:** Change of the area under cutoffs during the time of the study.

Cutoff number	Area of the cutoff in km^2^				Change and conversion (Observed in 2017)
	1932	1975	1995	2017	
1	0.080	-	0.049	0.030	Agricultural land and vegetation
2		-	0.06	0.008	Dried, marshy land and fish ponds
3	0.150	0.090	0.072	0.060	Agricultural land and fish pond
4		-	0.063	0.040	Agricultural land and marshy land
5	0.563	0.246	0.083	0.044	Marshy land, Agricultural land, and Fish ponds
6	0.205	0.110	0.041	-	Agricultural land
7		-	0.077	0.037	Agricultural land and Fish Pond
8	0.140	0.090	0.063	0.036	Agricultural land and Marshy land
9	0.090	0.020	0.037	0.032	Agricultural land and fish pond

The cutoffs were changed dramatically in size, with significant reductions from 1975 to 1995 and from 1995 to 2017. The cutoffs of 1932 are not mentioned in the Table 5 since there was a lack of correlation upon superimposing the other layers over it. Today, only cutoffs 3, 5, 6, and 9 of 1975 or earlier and cutoffs 1, 4, and 8 of 1995 can be found. All the cutoffs have shrunk considerably over time. However, cutoff 9, whose area appears to have increased in 1995 compared to 1975, cannot be justified. The satellite imagery was taken during the dry months when the cutoff had dried partially.

It is difficult to specifically attribute any reason for shrinking the cutoffs from 1975 to 1995 since the satellite data were not lucid enough. However, the 2017 conditions mostly feature their conversion to agricultural lands and fish ponds by the locals. A considerable number of cutoffs had also been transformed into marshes.

#### Condition of the cutoffs during the field visits

**Fig 7:**
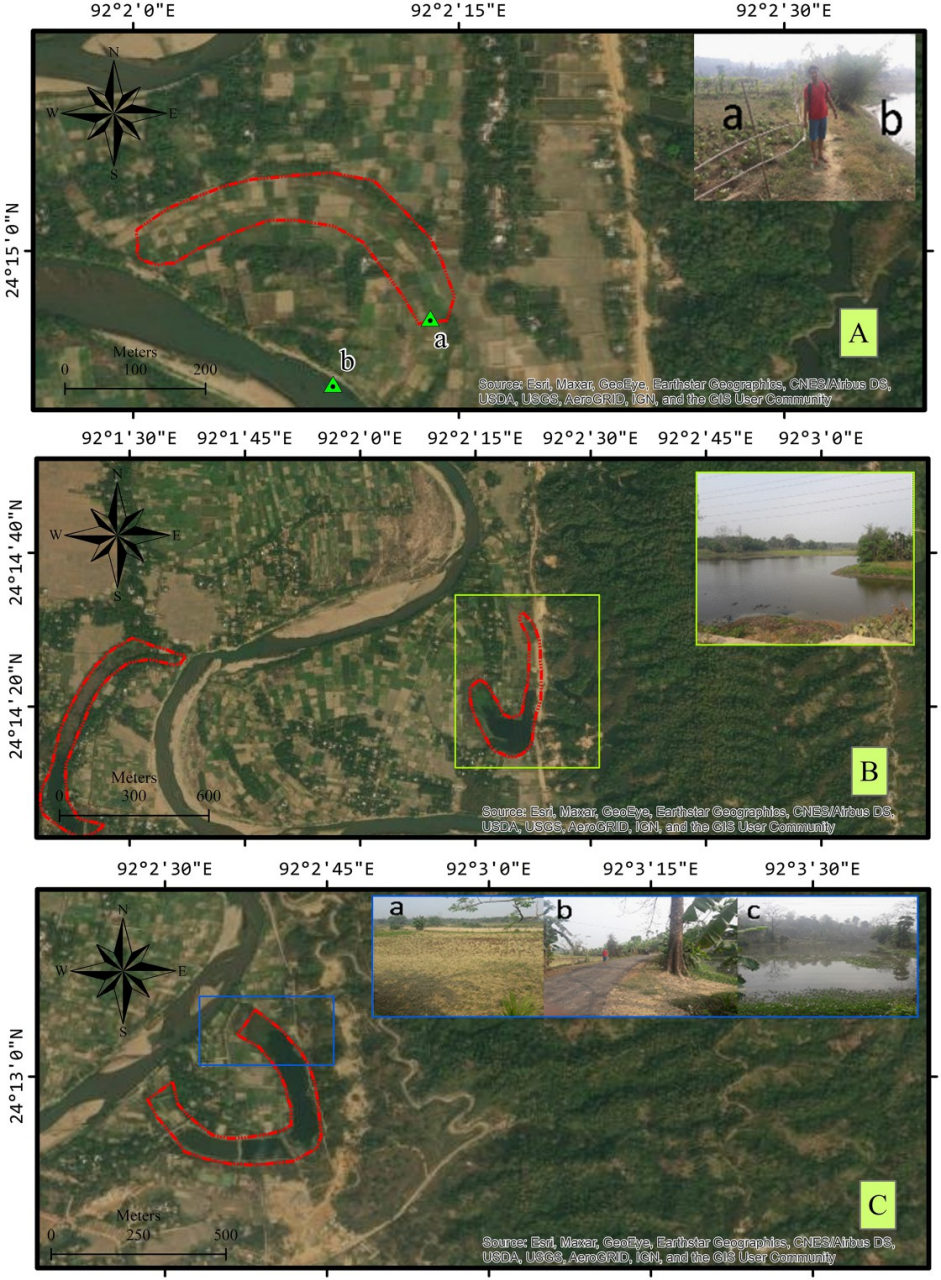
(A) Cutoff 6 at Jalai (a) Converted to agriculture (b) River Manu t1. (B) Cutoff 4 converted into marshy land. (C) Cutoff 3 near Naitingchhara (a) Part converted to agriculture (b) Road passing through the cutoff (c) Fish pond.

The area under every cutoff in 2017 was reduced compared to the earlier years of 1975 and 1995 (Table 5), largely due to human encroachment (Fig 7A–7C). A high inclination towards agriculture was observed. Owing to their fertility, after drying up or becoming transformed to marshes on the edges, the cutoffs eventually get converted into agricultural lands by the locals. All the selected cutoffs had been fully or partially converted to agricultural land in the study area, except cutoff 2. Apart from being used for agricultural purposes, the cutoffs were segmented and used as fish ponds, as seen in cutoffs 3, 5, 7, and 9. Furthermore, marshes had developed in cutoffs 2, 4, 5, and 8.

### Prediction of the future evolution of the meander

In the present study, five bends of River Manu, which have a high potentiality to form cutoffs in the event of significant floods in the coming years, were selected. They were bend 1 (Purba Ratacherra), bend 2 (Fatikroy), bend 3 (Jalai), bend 4 (near Srirampur) and bend 5 (Chhontail). To select the potential areas of new cutoffs, meander arm migration, bank erosion, bed scouring point, bank soil, and river water sediment load concentration were considered.

#### Assessment of bank erosion

**Fig 8:**
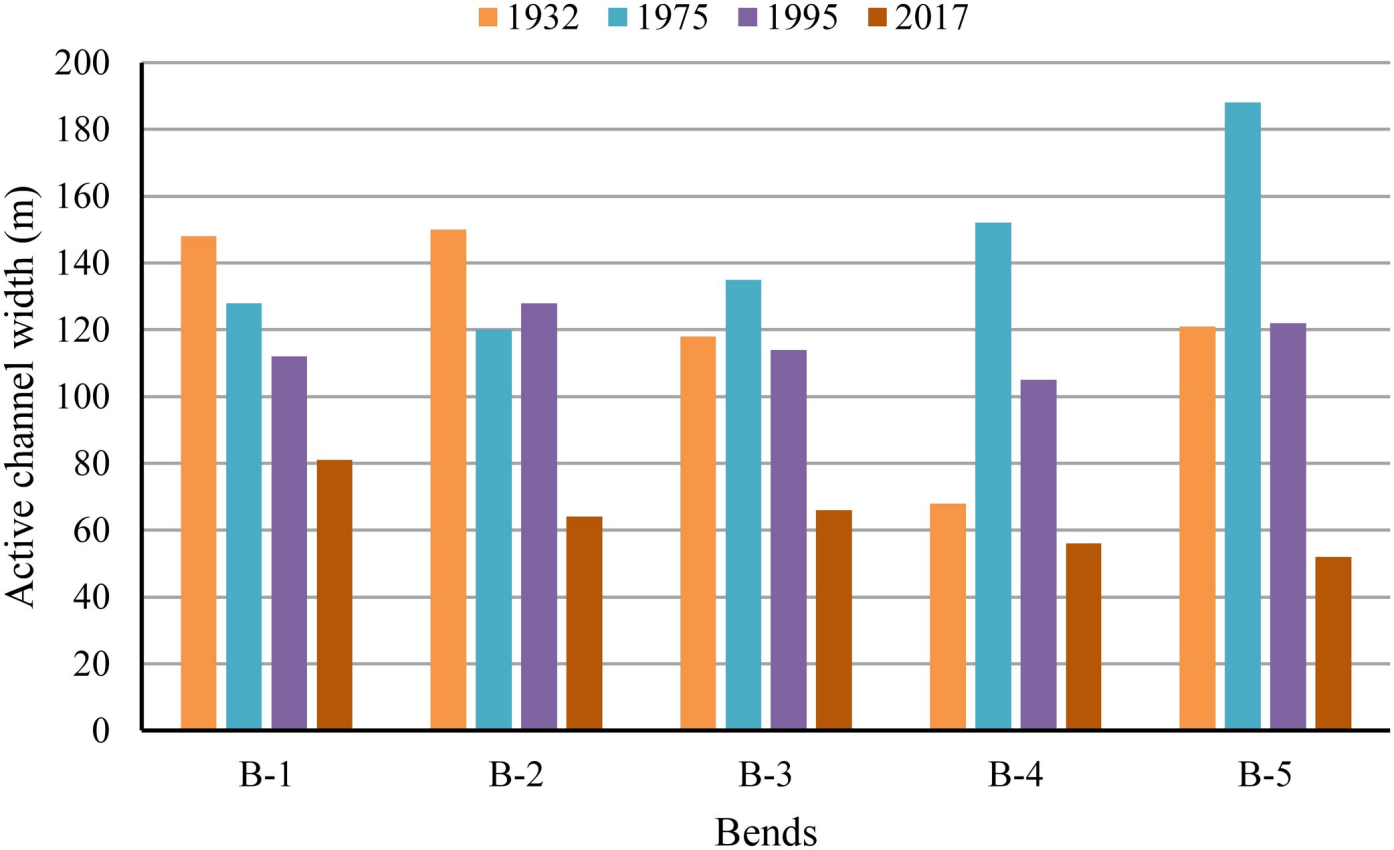
Width of River Manu for various years at the apex of selected five bends.

The width of a river channel increases as lateral erosion occurs along the banks. However, the width increased if the erosion rate was much higher than that of deposition. Fig 8 demonstrated that the width had increased considerably from 1932 to 1975 in bends 3, 4, and 5, while it decreased in bends 1 and 2. During 1995, the channel width decreased in all the selected bends, except bend 2. The active channel width again experienced a decreasing trend in all the selected bends in 2017, probably due to erosion lower than that in earlier years and lower flooding in the river. Moreover, the dam at Nalkata (upper course of the river) hinders the smooth water flow downstream, while boulders around the banks allow limited width expansion [50].

#### Migration of the river channel

**Fig 9:**
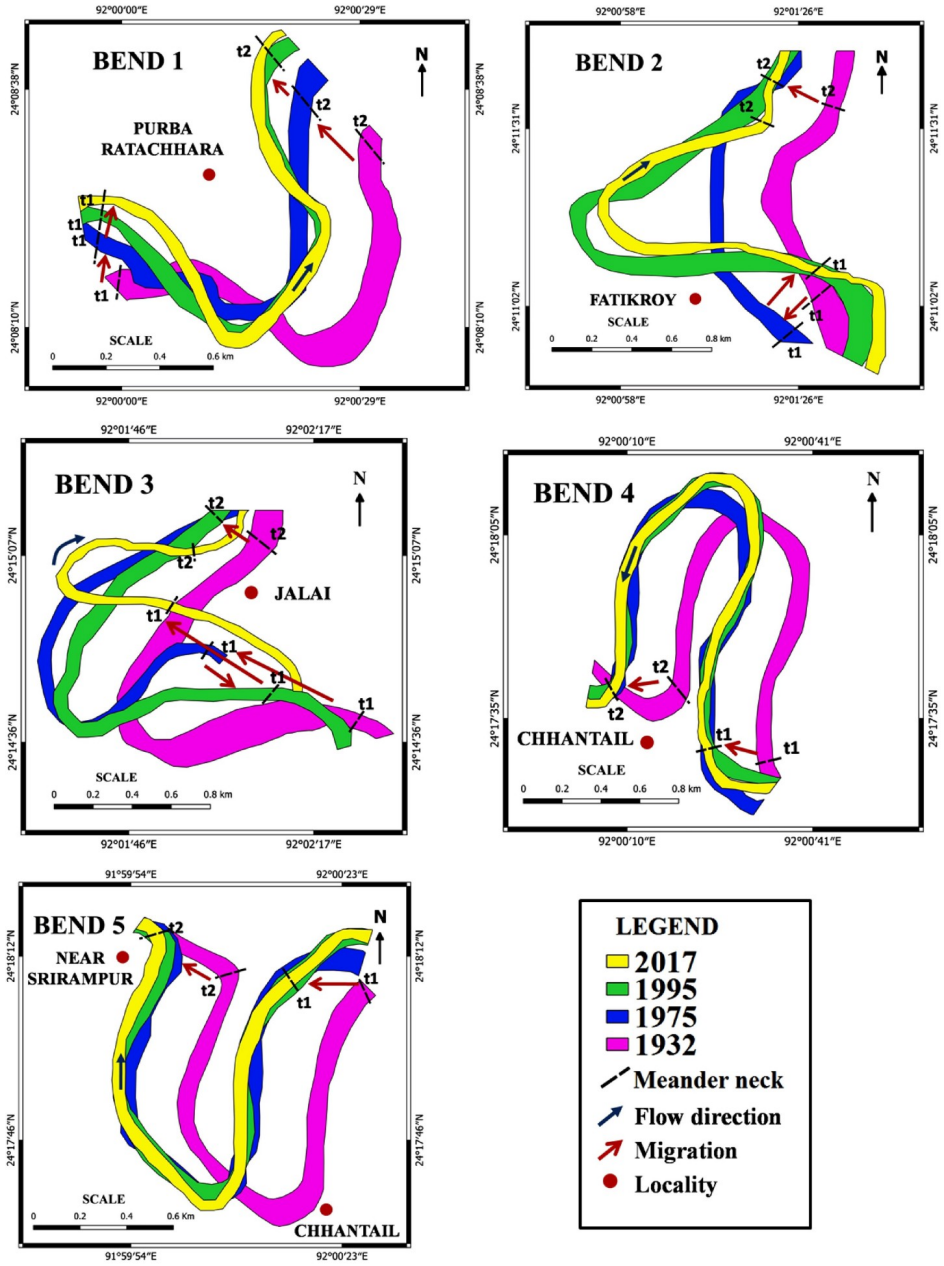
Migration of meanders for the years 1932, 1975, 1995, and 2017 at five selected bends.

The bend migration of the river was determined by comparing the images of the different years [51]. All the selected bends experienced significant channel meander shifts during the period 1932–1975, whereas only bends 1, 2, and 3 faced some shifting during the successive period, i.e., 1975–1995. The trend of lesser shifting prevailed during 1995–2017 (Table 6). In bend 1, the meander gradually shifted on the north-western side (Fig 9). The highest shifts of t1 and t2 were during 1932–1975, covering 86.1 m and 212.64 m, respectively.

**Table 6:** Historical migration of the selected meanders.

Bend	Migrated length in m					
	1932–1975		1975–1995		1995–2017	
	t1	t2	t1	t2	t1	t2
1	86.1	212.64	46.73	107.81	35.95	-
2	175.23	227.81	282.73	-	-	-
3	274.64	155.42	539.03	-	104.24	17.92
4	268.07	234.94	-	-	-	174.48
5	212.91	172.7	-	-	-	-

Conversely, in bend 2, the shifting occurred towards the eastern direction, and the highest shifting happened from 1975 to 1995. At this point, t1 shifted 282.73 m during the period 1975–1995, and t2 shifted 227.81 m during 1932–1975. The most significant shift was observed in bend 3 near Jalai, where the channel migrated from the southwestern to the western direction and finally narrowed the bend in 2017. Moreover, bends 4 and 5 showed significant change during 1932–1975.

#### Radius of curvature

The radius of curvature was reduced in all the bends from 1932 to 2017 (Table 7). Due to the narrowing of the meander, the radius decreased during the period 1932–1975 and again increased during the years 1995 and 2017 in bend 1. In bend 2, in 1975, a significant increase was found compared to that in 1932 as the meander wavelength increased. Later, the bend narrowed in 1995 and 2017. At bend 3, the maximum radius of curvature was observed during the year 1995, which again decreased in 2017. At bend 4, the radius increased from 1932–1995 but declined in 2017. In bend 5, the radius decreased during 1932–1975 but slightly broadened in 1995, followed by a decrease in 2017. As the radius decreases, the bend gets narrower and eventually favors the formation of new cutoffs.

**Table 7:** Radius of curvature of the selected bends.

Bend	Location	Radius in m			
		1932	1975	1995	2017
1	Purba Ratachhara	244.39	190.81	262.85	233.33
2	Fatikroy	281.2	609.52	179.48	196.59
3	Jalai	164.46	154.22	204.34	138.21
4	Chhontail	212.43	242.4	247.73	208.44
5	Near Srirampur	234.51	226.34	252.21	239.35

#### Cross-section of the meander bends

**Fig 10:**
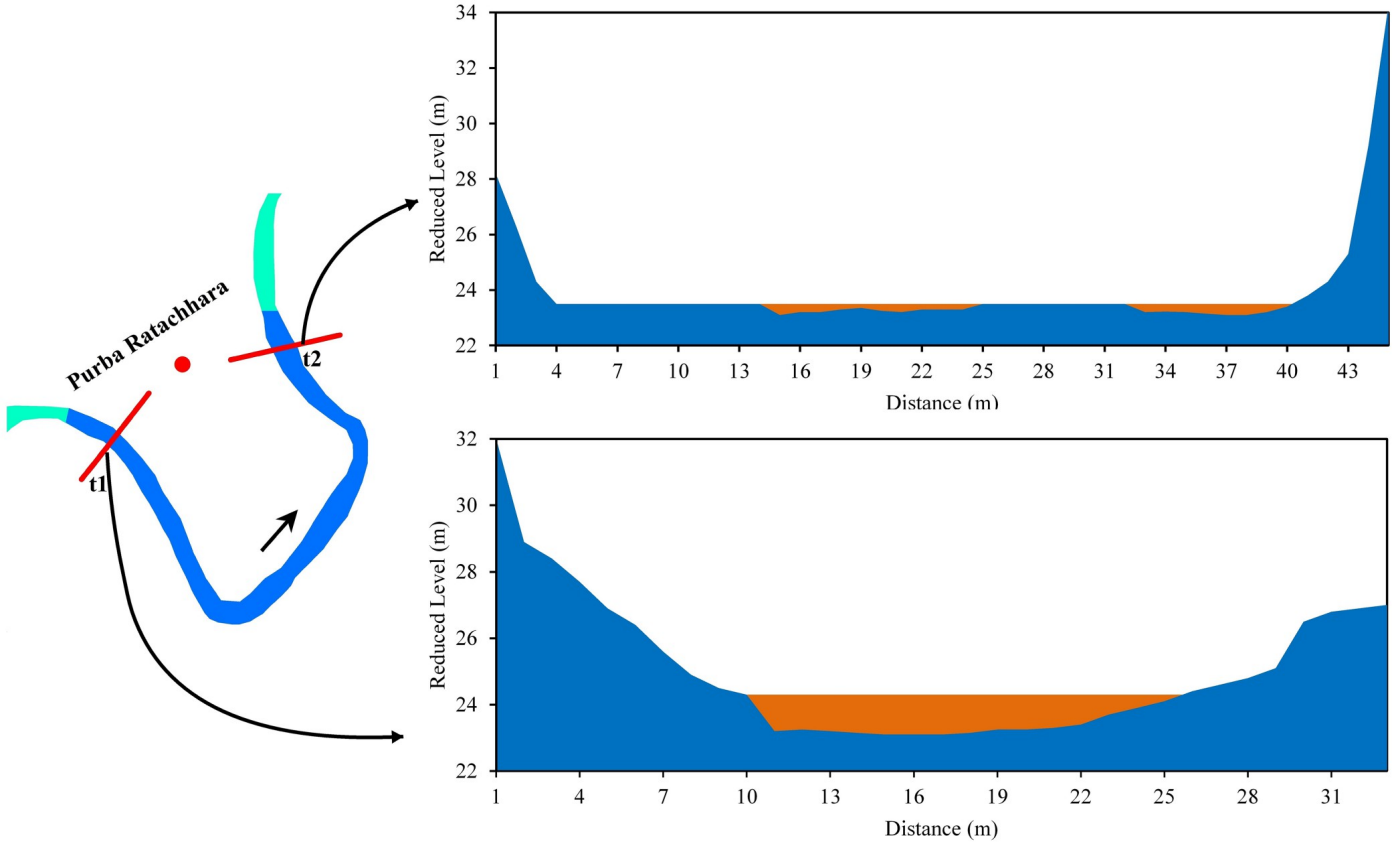
Cross-section across the Manu River at Purba Ratachhara (a) t1 (b) t2.

The cross-sectional area of the channel is a function of the mean depth and width along a transverse section of the stream. These two variables affect the discharge and hydraulic radius of a stream channel. Cross-sections were taken at the meander by considering the upstream section as t1 and the downstream section as t2. Ten cross-sections were taken in the five selected bends. In Purba Ratachhara, both t1 and t2 sections of the bend were asymmetrical. The river eroded laterally towards the left and right banks in both sections. The width of the channel at t1 was 22 m, with the greatest depth (0.3 m) in the left part of the river. The channel width at t2 was 72 m, with the greatest depth (0.3 m) in the left part of the river. Moreover, the river channel was shallow in the t2 section compared to the t1 section and had a mid-channel bar. The mean velocities at the t1 and t2 sections were 0.22 m/s and 0.25 m/s, respectively (Fig 10).

**Fig 11:**
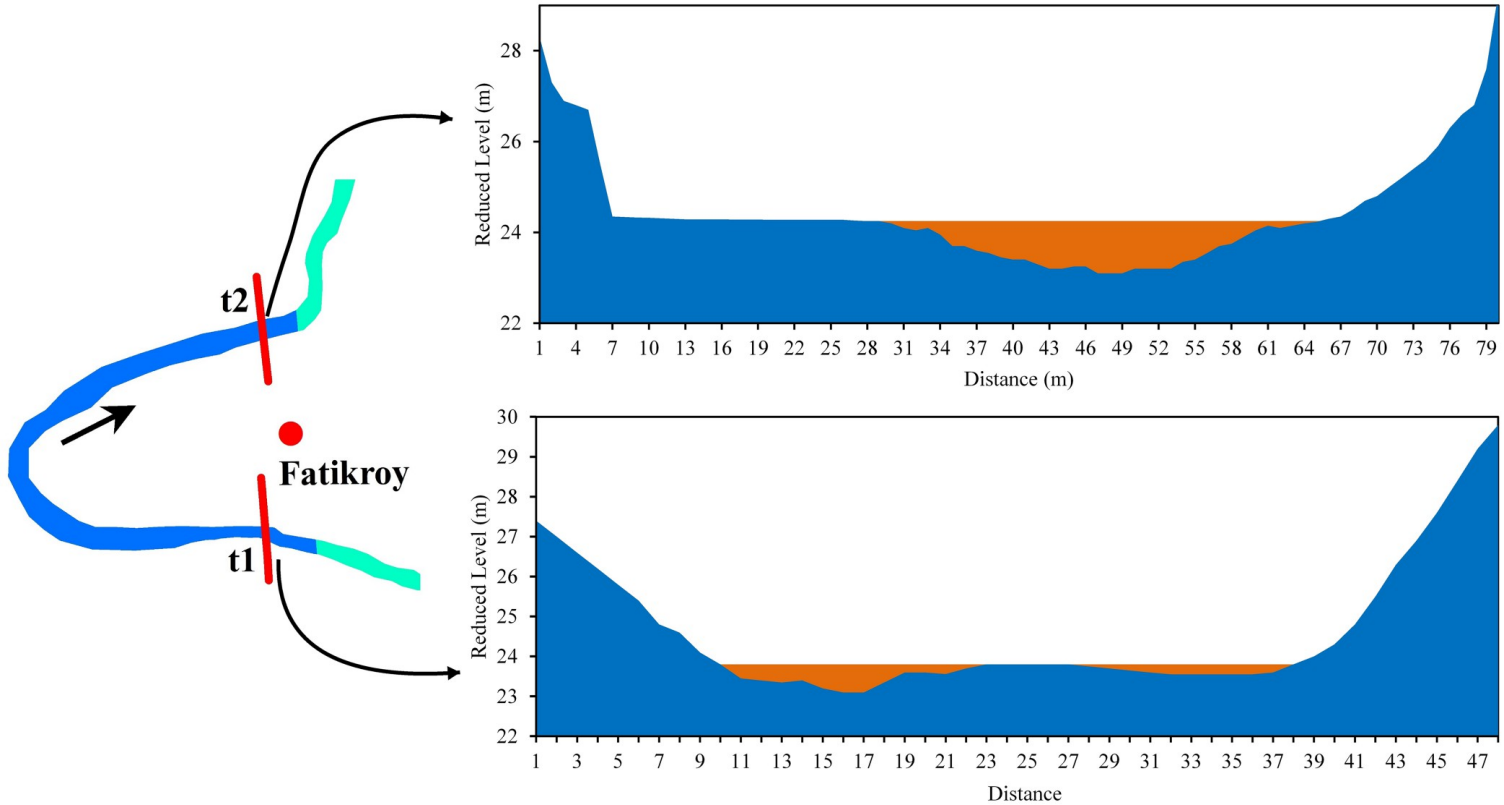
Cross-section across the Manu River at Fatikroy (a) t1 (b) t2.

In Fatikroy, the t1 section was symmetrical, but the t2 section was asymmetrical. Inward migration was observed at the neck of bend 2, which indicates the probability of a future cutoff. Both in the t1 and t2 sections of the bend, the right bank of the river was eroded laterally while deposition occurred at the left bank. The width of the river channel at t1 was 117.3 m, with the highest depth (1.2 m) in the central part of the channel. The width at t2 was 74 m, with the highest depth (0.7 m) in the left part of the river. At t2, the river had the bar in the middle, while in t1, the bar was on the left bank. The mean velocities at t1 and t2 were 0.08 m/s and 0.33 m/s, respectively (Fig 11).

**Fig 12:**
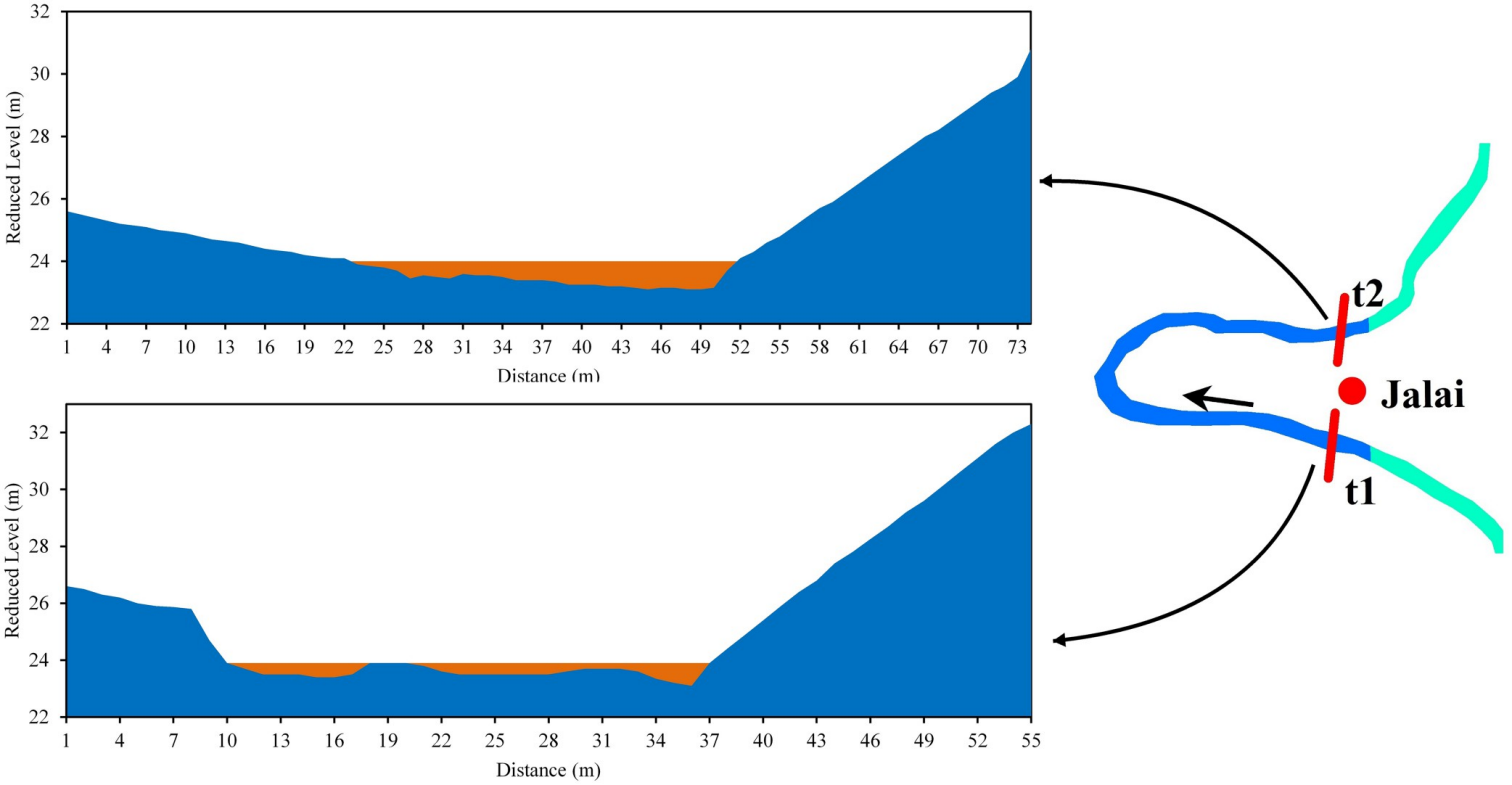
Cross-section across the Manu River at Jalai (a) t1 (b) t2.

In Jalai, both t1 and t2 sections of the bend were asymmetrical. In both sections, the river eroded laterally towards the right bank, and at t2, there was deposition on the left bank. The river’s width at t1 was 84 m, and the highest depth (0.8 m) was in the right part. The width at t2 was 56 m, with the highest depth (0.45 m) in the right part of the river. At t1, the river had the bar in the middle, while in t2, the bar was on the left bank. The mean velocities at t1 and t2 were 0.40 m/s and 0.37 m/s, respectively (Fig 12).

**Fig 13:**
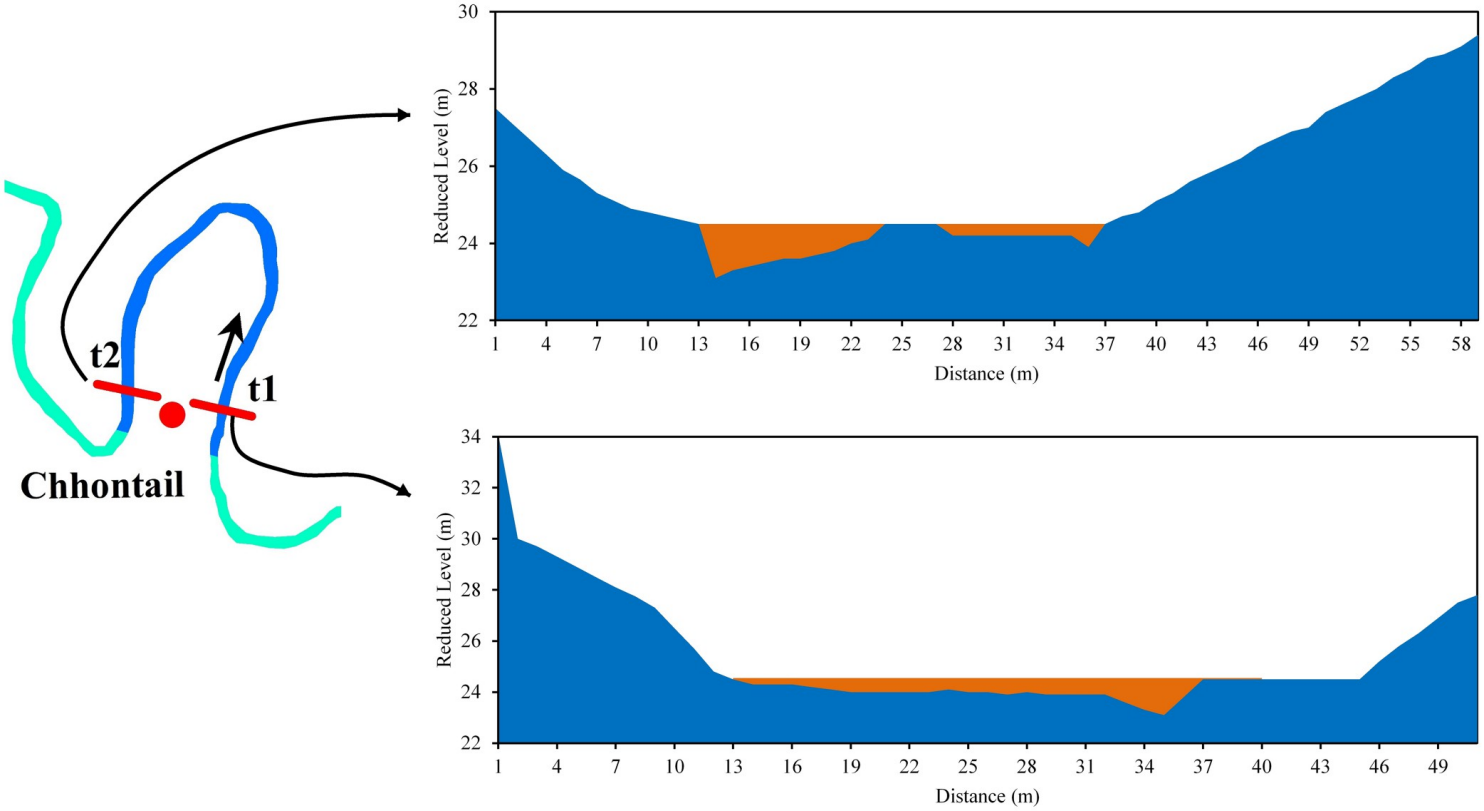
Cross-section across the Manu River at Chhontail (a) t1 (b) t2.

In Chhontail, the t1 and t2 sections of the bend were asymmetrical. In both sections of the river, the bend eroded the left bank laterally. The width of the river at t1 was 65 m, and the greatest depth (1.4 m) was in the left part. The width at t2 was 71 m, with the highest depth (1.3 m) in the right part of the river. In the t2 section, the river had the bar in the middle and right part, while in t1, the bar was on the right bank. The mean velocities at t1 and t2 were 0.21 m/s and 0.24 m/s, respectively (Fig 13).

**Fig 14:**
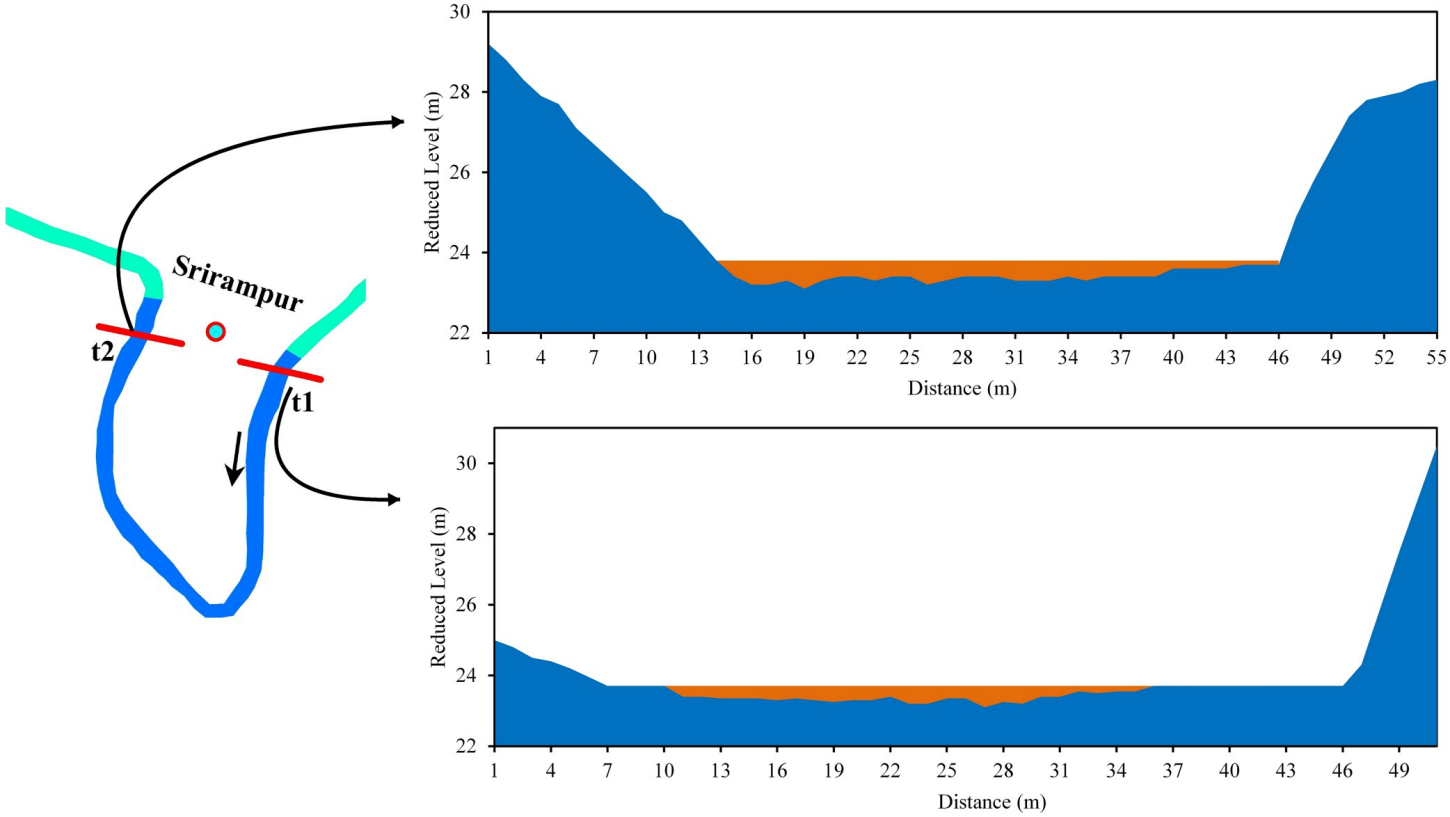
Cross-section across the Manu River at Srirampur (a) t1 (b) t2.

On the other hand, near the Srirampur bend, both the t1 and t2 sections of the river bend experienced lateral erosion towards the right bank, suggesting a future neck cutoff. The width of the river at t1 was 64 m, and the highest depth (0.7) was in the left part. The width at t2 was 78 m, with the highest depth (0.55 m) in the middle part of the river. The mean velocities at t1 and t2 were 0.20 m/s and 0.16 m/s, respectively (Fig 14).

#### Grain size distribution

Grain size is also called particle size and refers to the diameter of individual grains of the sediment. A standard method to determine the grain size is a sieve analysis or gradation test. Soil samples were collected from the upper and lower parts of the laterally eroding banks of the meandering arm (t1 and t2). Sieve analysis was performed by taking 100 g of that soil sample. According to the Indian Standard Soil Classification System, fine-grained soils are those in which >50 percent of the material has particle sizes <0.075 mm. The fine sand size of coarse soil ranges from 0.075 to 0.425 mm, and the medium sand size of coarse soil ranges from 0.425 to 2 mm (Table 8).

**Table 8:** Grain size classification by Indian Standard Soil Classification System.

**Very coarse soils**	Boulder size	-	>300 mm
	Cobble size	-	80–300 mm
**Coarse soils**	Gravel size (G)	Coarse	20–80 mm
		Fine	4.75–20 mm
	Sand size (S)	Coarse	2–4.75 mm
		Medium	0.425–2 mm
		Fine	0.075–0.425 mm
**Fine soils**	Silt size (M)	-	0.002–0.075 mm
	Clay Size (C)	-	< 0.002 mm

**Fig 15:**
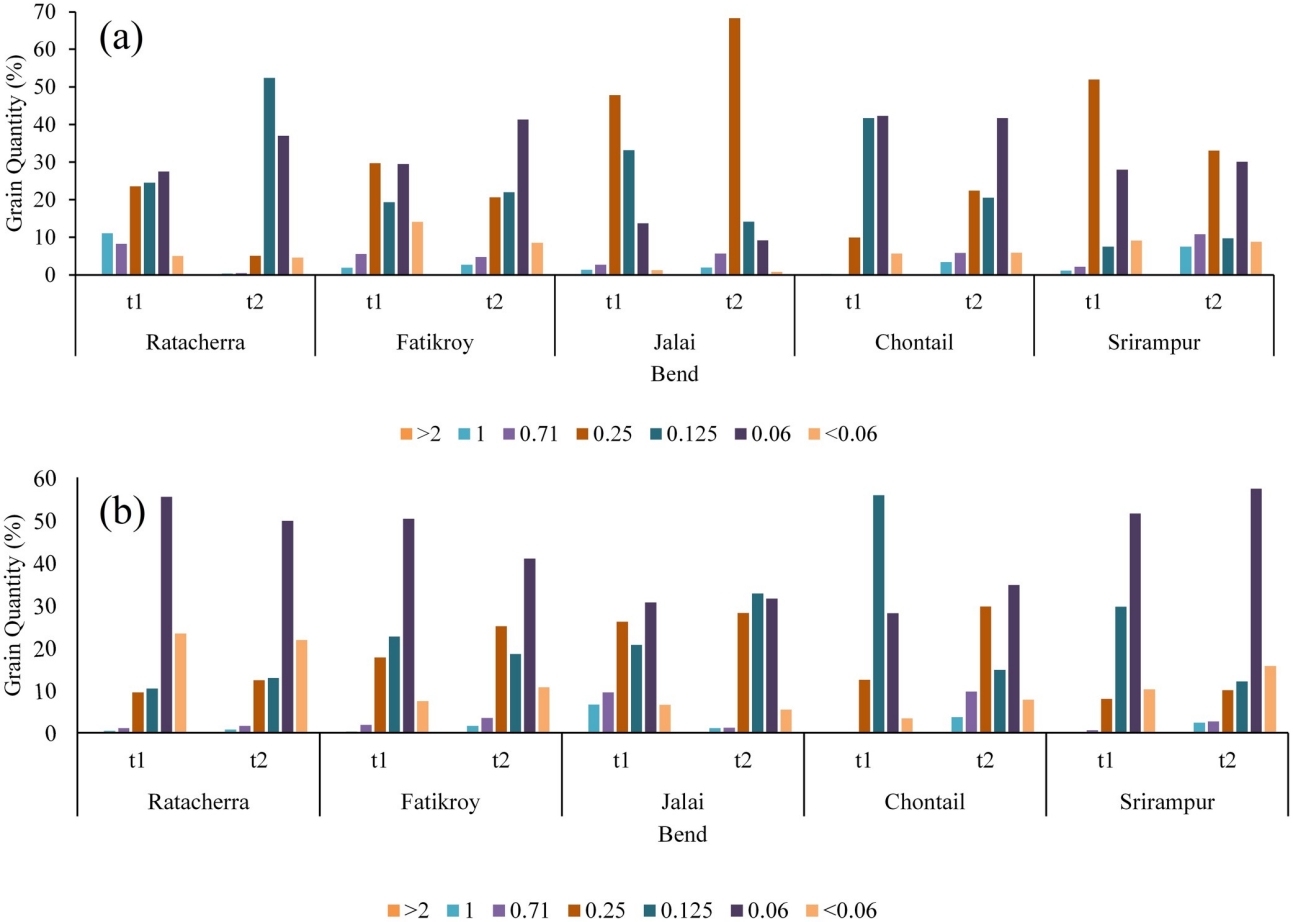
Grain size distribution per 100 grams in the upper and lower bank of the selected bends.

Fig 15 shows that the upper part of the riverbank mainly consists of coarse soil with fine sand (grain size 0.25–0.125 mm), whereas the lower part is chiefly made up of fine particles (grain size 0.06 mm). Fine particles include silt (0.002–0.075 mm) and clay (<0.002 mm), which are cohesive and, therefore, resistant to the force of water. On the other hand, soils with loose materials and particularly large grains are porous and easily eroded by the water. Well-graded soils have Cu ≥ 6 and 1≤ Cc ≤ 3. Soil gradation is important in geotechnical engineering. Poorly graded soil with high porosity and permeability is susceptible to soil liquefaction [52, 53]. This kind of soil does not have a good representation of all particle sizes from no. 4 to no. 200 sieve. Generally, the soil of River Manu is poorly graded (Table 9). In such conditions, river banks were easily susceptible to erosion during floods. The width of the river at the bends was higher than the other parts of the river, which may probably be due to heavy bank erosion influenced by sandy and poorly graded soil.

**Table 9:** Grain size analysis of the collected soil samples of the Manu Riverbank.

Bend	Part of bank	% Of passing in sieve #200 (0.075mm)	C_u_	C_C_	Remarks
Purba Ratachhara, t1	up	80.66	4.28	0.64	Poorly graded
	down	98.52	2.77	1.20	Poorly graded
Purba Ratachhara, t2	up	99.13	2.43	0.93	Poorly graded
	down	97.75	2.89	1.18	Poorly graded
Fatikroy, t1	up	92.60	4.78	0.81	Poorly graded
	down	97.93	2.18	0.91	Poorly graded
Fatikroy, t2	up	92.5	2.93	0.77	Poorly graded
	down	95.02	3.16	0.78	Poorly graded
Jalai, t1	up	95.93	3.59	0.89	Poorly graded
	down	83.83	4.30	0.62	Poorly graded
Jalai, t2	up	92.36	3.92	1.36	Poorly graded
	down	97.77	3.07	0.83	Poorly graded
Chhontail, t1	up	99.56	2.42	0.88	Poorly graded
	down	99.76	2.50	1.04	Poorly graded
Chhontail, t2	up	90.64	3.01	0.72	Poorly graded
	down	86.78	4.58	0.54	Poorly graded
Near Srirampur, t1	up	96.63	6.22	0.49	Poorly graded
	down	99.24	2.07	0.99	Poorly graded
Near Srirampur, t2	up	82	2.61	1.00	Poorly graded
	down	95	2.42	1.16	Poorly graded

**Fig 16:**
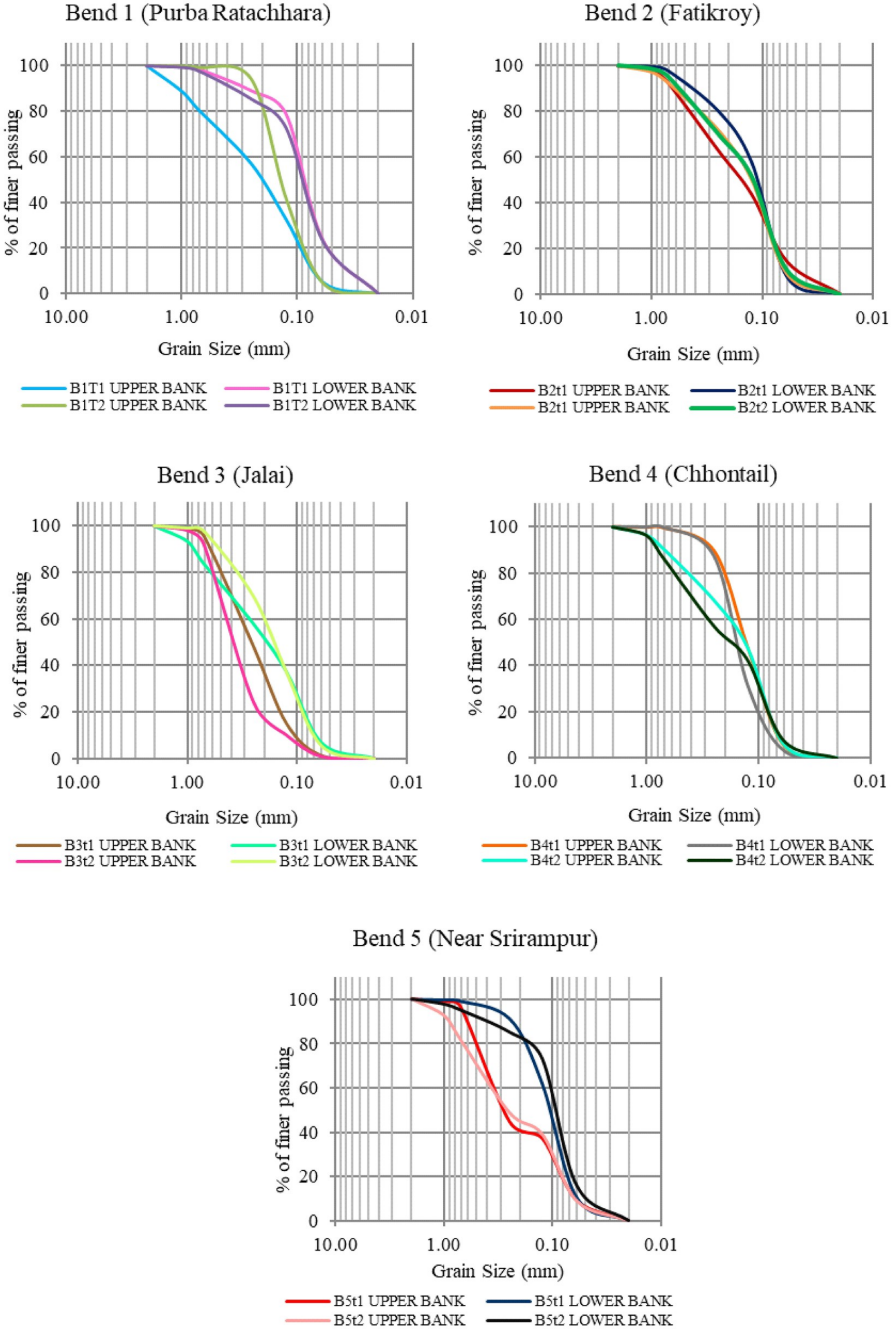
Grain size distributions using USCS in upper and lower bank soil of stations t1 and t2 for the five selected bends.

Overall, the soil in the study area was fine in nature (Fig 16), with the upper bank predominantly comprising sandy soil together with silt and the lower bank consisting of fine soil with a high percentage of silt. Fine soil, such as silt, is cohesive in nature; hence, it is resistant to water pressure in case of erosion. On the other hand, the upper bank with sandy soil is less resistant to erosion. Therefore, when the water rises during floods, there is a probability for the upper bank to crumble.

#### Suspended sediment concentration

**Fig 17:**
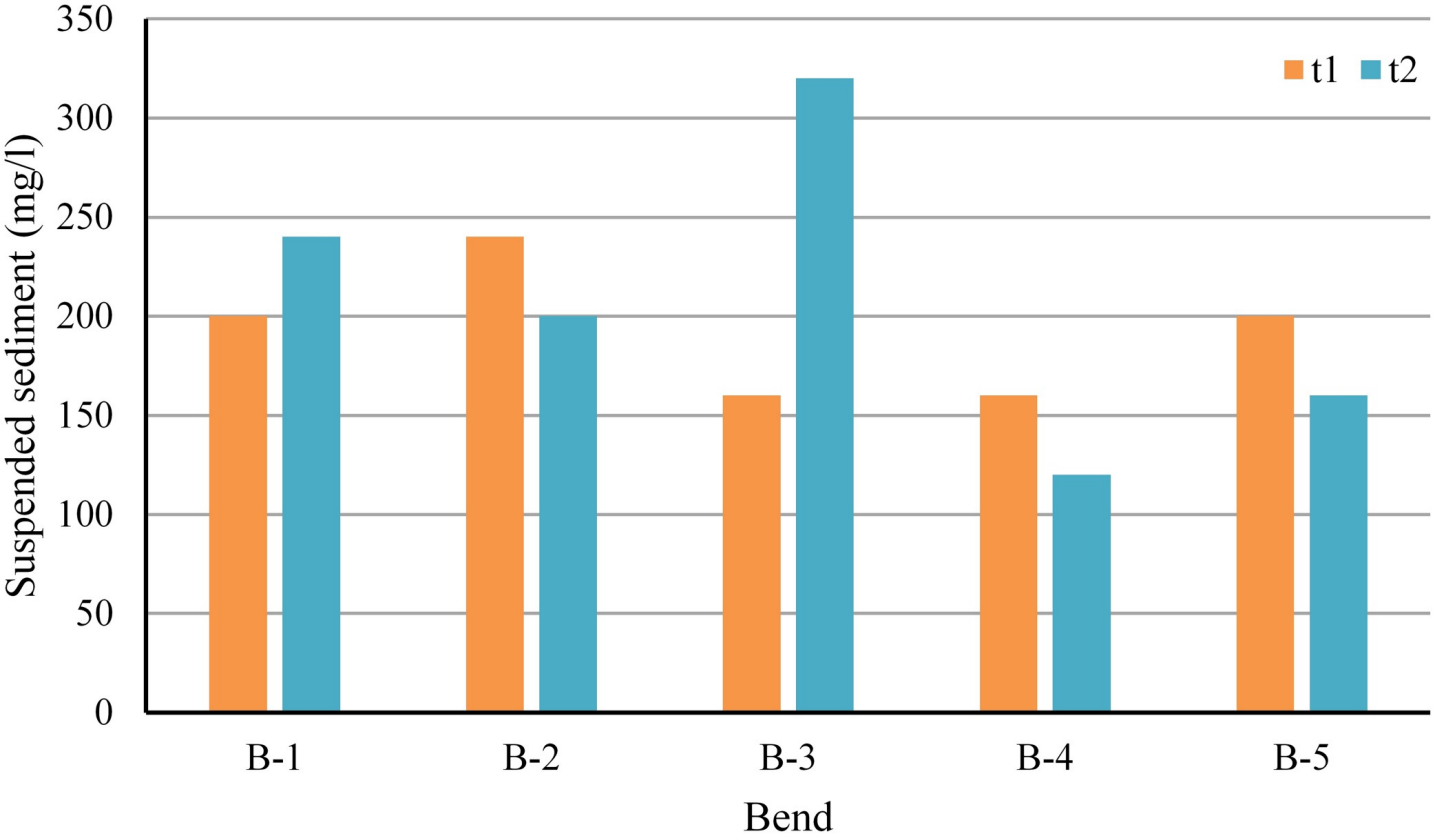
Suspended sediment concentrations in water at different bend sites.

The term ’suspended sediment concentration ’can be defined as ’the ratio of the mass of dry sediment in the water to the mass of the water-sediment mixture typically expressed in milligrams of dry sediment per liter of the water-sediment mixture. The results revealed that sediment concentration at Purba Ratachhara (Bend 1), Fatikroy (Bend 2), and Jalai (Bend 3) was 240 mg/litter, 200 mg/liter, and 320 mg/liter, respectively. Whereas, in the downstream region, i.e., Chhontail (Bend 4) and near Srirampur (Bend 5), it was 120 mg/liter and 160 mg/liter, respectively (Fig 17). In general, water had very little turbidity, probably because sampling was done before the arrival of the rainy season. Moreover, the Nalkata barrage present upstream could have disrupted the flow of the sediments.

#### Suspended sediment discharge

**Fig 18:**
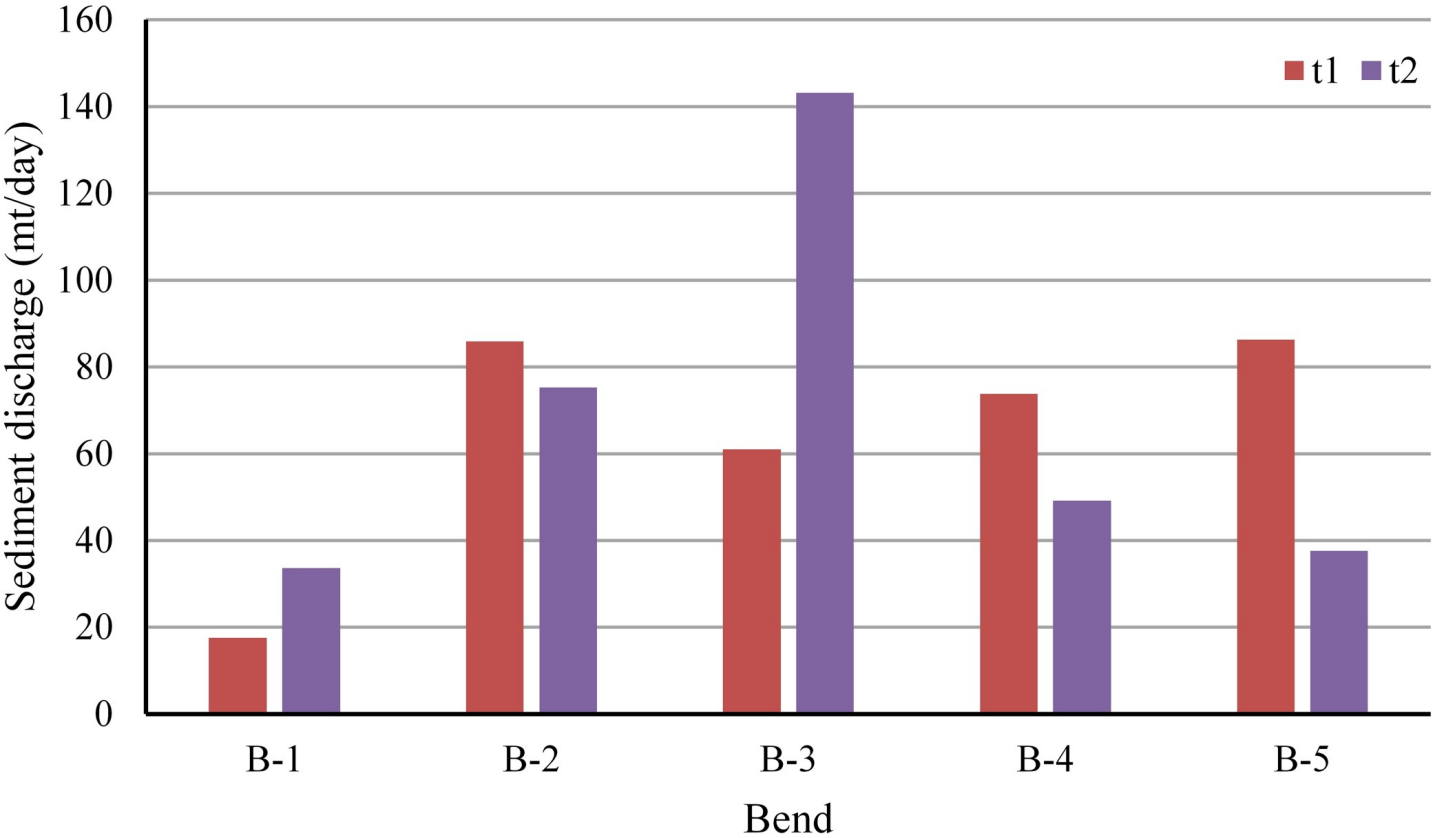
Suspended sediment discharge in the selected bends.

The suspended sediment discharge was 17.56 mt/day (t1) and 33.6 mt/day (t2) in Purba Ratachhara. In all the bends, the southern station t1 had higher discharge than the northern station t2, except for Purba Ratachhara and Jalai. Overall, Purba Ratachhara had less sediment discharge, which could be explained by its proximity to the Nalkata barrage that disrupts the flow. In Jalai, t2 had more discharge, which could be attributed to the greater width of the stream when compared to the width at t1. In almost all the bends, a decrease in suspended sediment from t1 to t2 was observed, which could be due to the deposition of these sediments in the inner side of the bend (point bars) while traveling from t1 to t2 station in the bend (Fig 18).

#### Potential areas of new cutoffs

**Fig 19:**
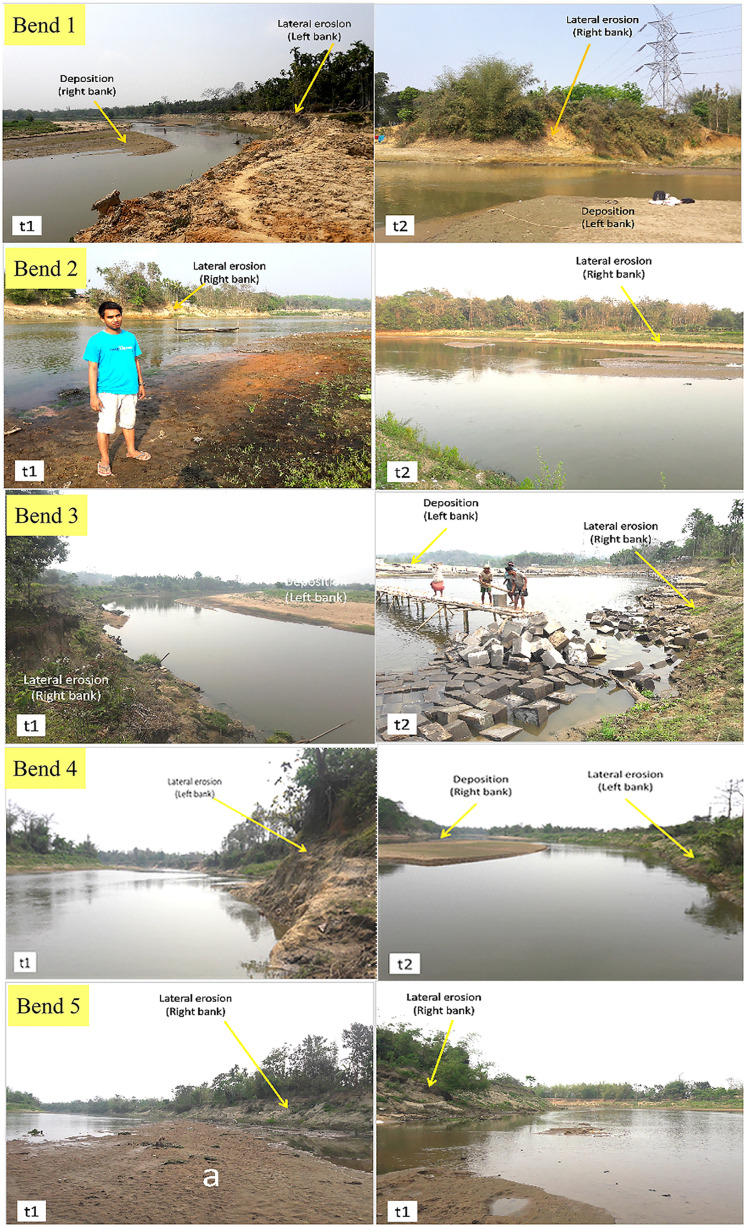
The present condition of banks of the five surveyed bends.

After studying the five selected bends, several inferences were made by applying superimposition techniques, meander geometry, and stream hydraulics. To justify the increasing probability of forming a cutoff in one meander, factors such as bend lateral erosion, bed scouring, presence of loose bank materials, and less vegetation in the same bank of the t1 (upstream) and t2 (downstream) sections were considered [54, 55]. The present conditions of the bends are shown in Fig 19 through field photographs.

**Fig 20:**
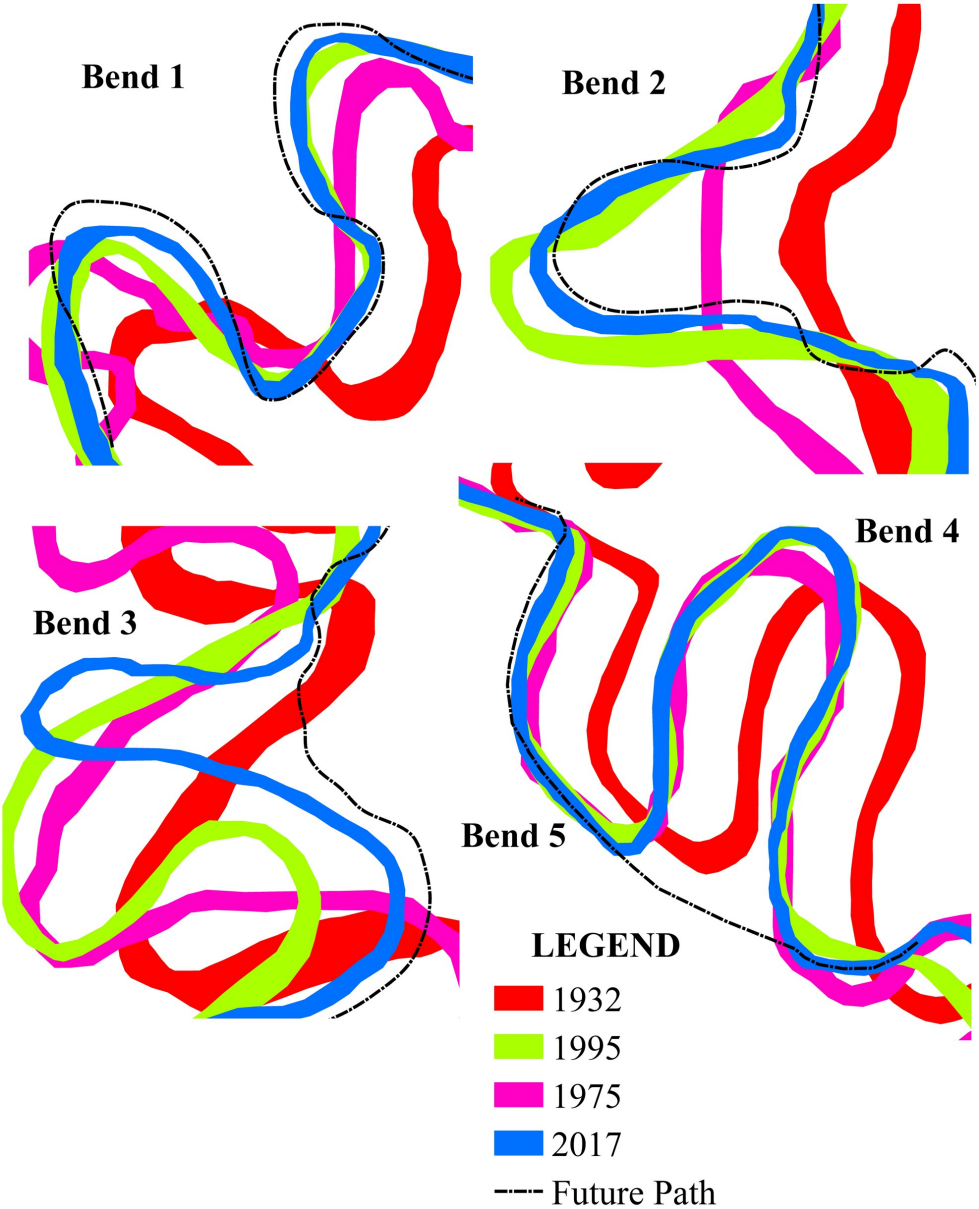
The probable future path of the selected meanders.

*Bend 1 (Purba Ratachhara)*. In the Purba Ratachhara bend, a cutoff is unlikely to be formed since the river was eroding laterally in the left bank in the t1 section of the bend, while the opposite was true for t2, in which deposits were made in the left bank. Erosion was rapidly taking place in the left bank at the t1 section as the mean velocity was higher than in the right bank. Moreover, water depth was larger at the left bank due to bed erosion (Fig 10). The maximum grain size of the bank soil was 0.25 and 0.125 mm (fine sand) in the upper part of the bank, and suspended sediment discharge was 17.56 mt/day (Fig 15). Therefore, the likelihood of forming a cutoff increases only in the case of heavy floods. Such coarse soils are loose and have high water permeability. On the other hand, the soil was much finer in the lower part of the bank. The radius of curvature also decreased to 0.21 m from 1995 to 2017, increasing the probability of cutoff formation (Fig 20, Bend 1).

*Bend 2 (Fatikroy)*. Inward migration was observed at the neck of bend 2, which suggests a future cutoff. In both the t1 and t2 sections of the bend, the river was eroding laterally in the right bank, and deposition was taking place in the left bank (Fig 20, Bend 2). However, in both cases, the velocity was more in the left part of the river channel, suggesting faster transport of sediments on the left bank, which was against the cutoff formation. Moreover, the radius of curvature had increased to 23 m from 1995 to 2017. It is also well known that if the meander loop area increases, the radius will increase; if the loop becomes too large, there are chances for a neck cutoff formation. Although the presence of maximum finer grain size and higher suspended discharge in the lower bank soil indicates a lesser probability for cutoff formation, the inward migration of the two arms of the meander signifies the chances for future cutoff. Furthermore, the fine sandy texture of the soil in the upper bank and the instability of the bank due to low vegetation favors cutoff formation in bend 2 [26, 56, 57].

*Bend 3 (Jalai)*. The change in channel planform and hydraulic parameters indicates the high probability of future cutoff at bend 3. In both the t1 and t2 sections of the bend, the river channel was eroding laterally on the right bank. At t2, there was deposition in the left bank (Fig 20, Bend 3). The depth was more (1 m) in the right part compared to the channel’s other parts (Fig 12). The mean velocity in the right part of the t1 and t2 sections of the river was 0.44 m/s and 0.40 m/s, respectively. At the same time, the mean velocity was 0.15 m/s and 0.10 m/s, respectively, in the left part of the sections. The radius of curvature had decreased with a considerable margin of 66.3 m from 1995 to 2017. Although sediment discharge was more, the presence of coarser particles in the bank soil and the rapid inward migration of the meander arms indicate a greater probability of future cutoff.

*Bend 4 (Chhontail)*. The cutoff is likely to occur at bend 4 in the near future. In both the t1 and t2 sections of the river bend, the left banks were undergoing lateral erosion, suggesting a neck cutoff. In both the sections, deposition took place in the right bank (Fig 20, Bend 4). The radius of curvature had decreased to 39.2 m from 1995 to 2017, thus narrowing the loop area. Therefore, it could be easily concluded that narrow loop area, inward arm migration, coarser grain material with low vegetation in the bank, and low sediment discharge imply chances for future cutoff.

*Bend 5 (near Srirampur)*. Cut off in bend five is possible in case of frequent heavy floods. The right banks (t1 and t2) were observed to have been eroded, although there was sediment deposition at certain points (Fig 20, Bend 5). It suggests that erosion occurred in this part at times of flood only when the water level was increased. Moreover, in both the sections, the depth and velocity were higher in the right part than in other parts of the river channel (Fig 14). The radius of curvature was also narrower than that of the previous year. These factors shed light on the chances of the cutoff at bend 5. Besides, the presence of coarser grain with lower vegetation in the bank and lesser sediment discharge indicates the possibilities for future cutoff.

### Future migration of meanders

Based on the migration pattern of the past years, we can predict the likely future paths. Also, the observation of the erosion patterns, as seen in the cross-section, helps us make logical assumptions regarding the future migration paths of the river. Bend 1 is likely to move further in the west, with a narrowing down of the radius of curvature. Bend 2 is expected to move to the west. Bend 3 and 4 may straighten up the channel by leaving a cutoff. Bend 5 is likely to straighten up the channel, although it is hard to say with certainty since these bends have shown significantly less migration in the past (Fig 20). It is difficult to predict the future of meanders precisely. Small insights can be gained by examining the past trends and the significant changes in the present, which can contribute to the future development of meanders. Such changes may be in the form of future cutoff, channel shift, or decrease in the width. In the present study, it was observed that bends 3 and 4 have the potentiality of forming a cutoff due to their lateral erosion inward and deposition outward. In contrast, bends 1, 2, and 5 may go through the cutoff process in case of major floods.

Moreover, the depth was less (0.40 m on average) in all the cross-sections. At the time of the field study (dry month, April 2018), the sediment discharge had an average of 66.34 mt/day, which is insufficient to bring significant changes in meanders. However, during peak discharges (rainy season), this river may impact erosion due to the acceleration of flow velocity. The riverbanks had a sandy texture of the soil on the upper bank and finer soil on the lower bank, which suggests more chances of erosion on the upper bank during times of flood when the water level rises. Moreover, bank instability due to less vegetation may also exacerbate the chances of lateral erosion, which may incite cutoffs when inward at the t1 and t2 sections.

There are various studies with similar works. Guo et al., 2019 [26] studied the skewing of bends and their relationship with the sinuosity in the Chulym River, Russia. This field-based study showed that without external forcings, meander bends tend to evolve from low-sinuosity bends that are downstream-skewed and transform to high-sinuosity bends that are upstream-skewed before the cutoff. These were the similar finding as observed in the present study, wherein all the field data from all the five bends showed similar observations. Further, without using numerical models, the study provided a means to interpret the bend evolution, similar in some ways to what has been conducted in the present study. Other studies have used numerical models to determine the evolution of river meandering. Asahi et al., 2013 [27] used river meandering processes such as bank erosion, land accretion, and channel cutoff formation to develop a complex computational model to evaluate various riverbank processes quantitatively. The model evaluated the depositional and erosional processes to understand the bed morphodynamics and used that to propose the relationship between sinuosity and river width. Further, various other studies have used numerical models to understand the meandering processes and their role in the sediment supply. In this regard, Inoue et al., 2020 [58] developed used a numerical model to reproduce the flooding events of 2010 and 2016 that destroyed the Kyusen Bridge over the Bebetsu River, Hokkaido, Japan. This study further suggested a strong relationship between erosional bank rates, flow discharge, and sediment supply, as was observed in our study as well.

## Conclusion

The present work has dealt with the temporal evolution of meandering rivers. From the present study, through spatio-temporal and hydrological analyses, we can conclude that paleo channels, such as cutoffs, are the live signatures of meandering channel migration. The increase in the number of cutoffs and the SI suggests the straightening of the channel. This straightening can be further inferred from the decrease in the number of meanders and the channel length. Meanders have positively shifted towards the west. Both the width at the apex of the meanders and the radius of curvature have decreased substantially, hinting at potential cutoffs in some meanders. In the upper part of the bank, the maximum grain size is concentrated at 0.25 and 0.125, which is categorized under coarse soil with fine sand. Maximum grains are finer with a grain size of 0.06 mm, taking the lead in the lower part of the bank. This includes silt (0.002–0.075 mm) and Clay (< 0.002 mm). The high percentage of finer grain size in the bank soil indicates a lower soil erosion rate. Still, the small percentages of the coarser material significantly influence the bank of the meander, as observed during the field study. Moreover, the cu and cc of the grain range between 6.22 to 1.07 and 0.49 to 1.36, respectively. Hence, it indicates that the bank materials are poorly graded, leading to high bank erosion. The analysis of cross-section, sediment discharge, grain size analyses of the bank material, channel planform change, and radius of curvature suggests that almost all the selected bends have the probability of future cutoff. The mean velocities at the bed scoring site of every cross-section channel ranged from 0.20 m/s to 0.40 m/s. In contrast, depth ranged from 0.3 m to 1.4 m, higher than the other part of the channel. Considering the above factors, the greatest probability of future cutoff was found in bend 3 (Jalai) and bend 4 (Chhontail). The future migration of meanders is difficult to identify since it is governed by several parameters that keep on changing. The present study is a significant step toward comprehending the meanders using the remote sensing and GIS technique along with field observation. We understood the nature of meander migration and the bank’s stability. Further work in this field is required to provide deeper insights into the role of anthropogenic factors in aggravating the river meander process.

## Supporting information

S1 Table Cross section across the Manu River at Purba Ratachhara (t1). (DOCX)

S2 Table Cross section across the Manu River at Purba Ratachhara (t2). (DOCX)

S3 Table Cross section across the Manu River at Fatikroy (t1). (DOCX)

S4 Table Cross section across the Manu River at Fatikroy (t2). (DOCX)

S5 Table Cross section across the Manu River at Jalai (t1). (DOCX)

S6 Table Cross section across the Manu River at Jalai (t2). (DOCX)

S7 Table Cross section across the Manu River at Chhontail (t1). (DOCX)

S8 Table Cross section across the Manu River at Chhontail (t2). (DOCX)

S9 Table Cross section across the Manu River at Srirampur (t1). (DOCX)

S10 Table Cross section across the Manu River at Srirampur (t2). (DOCX)

S11 Table Grain size distribution per 100 grams in the upper and lower bank of the selected bends. (DOCX)

S12 Table Suspended sediment concentration in water at different bend sites. (DOCX)

S13 Table Suspended sediment discharge in the selected bends. (DOCX)

## References

[1] Eke E, Czapiga M, Viparelli E, Shimizu Y, Imran J, Sun T, et al(2014)Co-evolution of width and sinuosity inmeandering rivers. J Fluid Mech 760:127–174. doi: 10.1017/jfm.2014.556

[2] Frascati A, Lanzoni S (2009) Morphodynamic Regime and Long-Term Evolution of Meandering Rivers. J Geophys Res 114. doi: 10.1029/2008JF001101

[3] Das P (2012) Meandering nature of Barak River in subtropical climate of Southern Assam, Northeast India-A Geospatial analysis. Int J Env Sci 2(4):2110–2119. doi: 10.6088/ijes.00202030094

[4] Hooke JM (2007) Spatial variability, mechanisms and propagation of change in an active meandering river. Geomorphology 84: 277–296.

[5] Seminara G (2006) Meanders. J Fluid Mech554: 271–297.

[6] Li Z, Gao P (2019) Channel adjustment after artificial neck cutoffs in a meandering river of the Zoige basin within the Qinghai-Tibet Plateau, China. Catena 172:255–265. doi: 10.1016/j.catena.2018.08.042

[7] Leopold LB, Wolman MG (1960) River meanders. Geol Soc Am Bull 71:769–793.

[8] Dietrich WE, Smith JD (1983) Influence of the pointbar on flow through curved channels. Water Resour Res 19: 1173–1192.

[9] Allen JRL (1965) A review of the origin and characteristics of recent alluvial sediments. Sedimentology 5:89–191. doi: 10.1111/j.1365-3091.1965.tb01561.x

[10] Ward JV, Tockner K, Arscott DB, and Claret C (2002) Riverine landscape diversity. Freshw Biol 47:517–539.

[11] Madej MA, Weaver WE, Hagans DK (1994) Analysis of bank erosion on the Merced River, Yosemite Valley, Yosemite National Park, California, USA. Environ Manag 18(2):235–250. doi: 10.1007/BF02393764

[12] Hooke JM (2013) River Meandering. Academic Press, San Diego, CA.

[13] Li Z, Wu X, Gao P (2019) Experimental study on the process of neck cutoff and channel adjustment in a highly sinuous meander under constant discharges. Geomorphology 327:215–229 doi: 10.1016/j.geomorph.2018.11.002

[14] Dieras PL (2013) The Persistence of Oxbow Lakes as Aquatic Habitats: An Assessment of Rates of Change and Patterns of Alluviation. Thesis submitted for the degree of Doctorate of Philosophy.

[15] Stølum HH (1996) River meandering as a selforganization process. Science 271: 1710–1713.

[16] Camporeale C, Perucca E, Ridolfi L (2008) Significance of cut-off in meandering river dynamics. J Geophy Res 113. doi: 10.1029/2006JF000694

[17] Zinger JA, Rhoads BL, Best JL (2011) Extreme sediment pulses generated by bend cutoffs along a large meandering river. Nat Geosci 4(10):675–678. doi: 10.1038/ngeo1260

[18] Eekhout JPC, Hoitink AJF (2015) Chute cutoff as a morphological response to stream reconstruction: The possible role of backwater. Water Resour Res 51:3339–3352. 10.1002/2014WR016539.

[19] Hooke JM (1995) River channel adjustment to meander cutoffs on the River Bollin andRiver Dane, northwest England. Geomorphology 14:235–253. doi: 10.1016/0169-555X(95)00110-Q

[20] Camporeale C, Perona P, Porporato A, Ridolfi L (2005) On the long-term behavior of meandering rivers. Water Resour Res 41(12):1–13. doi: 10.1029/2005WR004109

[21] Słowik M (2016) The influence of meander bend evolution on the formation of multiple cutoffs: findings inferred from floodplain architecture and bend geometry. Earth Surf Process Landf 41:626–641. doi: 10.1002/esp.3851

[22] Viero DP, Dubon SL, Lanzoni S (2018) Chute Cut-offs in Meandering Rivers: Formative Mechanisms and Hydrodnamic Forcing. International Association of Sedimentologists (IAS Special Publications).

[23] Annayat W, Sil BS (2020) Assessing channel morphology and prediction of centerline channel migration of the Barak River using geospatial techniques. Bull EngGeol Environ 79:5161–5183. doi: 10.1007/s10064-020-01894-9

[24] Deb M., Ferreira C. (2015) Planform channel dynamics and bank migration hazard assessment of a highly sinuous river in the north-eastern zone of Bangladesh. Environ Earth Sci 73:6613–6623. doi: 10.1007/s12665-014-3884-3

[25] Pourbakhshian S., Pouraminian M. (2015) Stochastic modeling to prediction of river morphological changes. Ind J Sci Tech 8(12):1–10. doi: 10.17485/ijst/2015/v8i11/71772

[26] Guo X, Chen D, Parker G (2019) Flow directionality of pristine meandering rivers is embedded in the skewing of high-amplitude bends and neck cutoffs. Proceedings of the National Academy of Sciences 116(47):23448–54. doi: 10.1073/pnas.1910874116 31685630 PMC6876206

[27] Asahi K, Shimizu Y, Nelson J, Parker G (2013) Numerical simulation of river meandering with self-evolving banks. J Geophys Res Earth Surf 118: 2208–2229, doi: 10.1002/jgrf.20150

[28] Debnath J, Das (Pan) N, Sharma R, Ahmed I (2019) Impact of confluence on hydrological and morphological characters of the trunk stream: a study on the Manu River of North-east India. Env Earth Sci 78:190. doi: 10.1007/s12665-019-8190-7

[29] Kasvi E, Lamaneen L, Lotsari E, Alho P (2017) Flow Patterns and Morphological Changes in a Sandy Meander Bend during a Flood—Spatially and Temporally Intensive ADCP Measurement Approach. Water 9(2):106. doi: 10.3390/w9020106

[30] Guneralp I, Marston RA (2012) Process–form linkages in meander morphodynamics: bridging theoretical modeling and real world complexity. Prog Phys Geogr 36:718–746.

[31] Romshoo SA, Altaf S, Rashid I, Dar RA (2018) Climatic, geomorphic and anthropogenic drivers of the 2014 extreme flooding in the Jhelum basin of Kashmir, India. Geomatics, Natural Hazards and Risk, 9(1), 224–248.

[32] Altaf F, Meraj G, Romshoo SA (2013). Morphometric analysis to infer hydrological behaviour of Lidder watershed, Western Himalaya, India. Geography Journal, 2013.

[33] Kanga S, Meraj G, Farooq M, Nathawat MS, Singh SK. 2021 Analyzing the risk to COVID‐19 infection using remote sensing and GIS. Risk Analysis 41(5):801–13. doi: 10.1111/risa.13724 33733497 PMC8251091

[34] Kanga S, Meraj G, Farooq M, Nathawat MS, Singh SK. Risk assessment to curb COVID-19 contagion: A preliminary study using remote sensing and GIS. doi: 10.21203/rs.3.rs-37862/v1

[35] Meraj G. 2020 Ecosystem service provisioning–underlying principles and techniques. SGVU J. Clim. Chang. Water, 7:56–64.

[36] Meraj G, Farooq M, Singh SK, Islam M, Kanga S. 2021 Modeling the sediment retention and ecosystem provisioning services in the Kashmir valley, India, Western Himalayas. Modeling Earth Systems and Environment. 27:1–26.

[37] Rather MA, Meraj G, Farooq M, Shiekh BA, Kumar P, Kanga S, et al 2022. Identifying the Potential Dam Sites to Avert the Risk of Catastrophic Floods in the Jhelum Basin, Kashmir, NW Himalaya, India. Remote Sensing. 14(7):1538.

[38] Wani AA, Bali BS, Ahmad S, Nazir U, Meraj G. 2022 Geospatial Modeling in Landslide Hazard Assessment: A Case Study along Bandipora-Srinagar Highway, NW Himalaya, J&K, India. In Geospatial Modeling for Environmental Management (pp. 113–125). CRC Press.

[39] Altaf S, Meraj G, Romshoo SA. 2014 Morphometry and land cover based multi-criteria analysis for assessing the soil erosion susceptibility of the western Himalayan watershed. Environmental monitoring and assessment. 186(12):8391–412. doi: 10.1007/s10661-014-4012-2 25154685

[40] Meraj G, Romshoo SA, Yousuf AR, Altaf S, Altaf F. 2015 Assessing the influence of watershed characteristics on the flood vulnerability of Jhelum basin in Kashmir Himalaya. Natural Hazards. 77(1):153–75.

[41] Meraj G, Romshoo SA, Ayoub S, Altaf S. 2018 Geoinformatics based approach for estimating the sediment yield of the mountainous watersheds in Kashmir Himalaya, India. Geocarto International. 33(10):1114–38.

[42] Chakraborty S, Mukhopadhyay S (2015) Riverbank erosion and channel width adjustments across a meandering channel of North Bengal, India. Earth Sci India 8(3):61–78.

[43] Schumm SA (1963) Sinuosity of alluvial rivers on the Great Plains. Bull Geol Soc Am 74:1089–1100. doi: 10.1130/0016-7606(1963)74[1089:SOAROT]2.0.CO;2

[44] Brice JC (1954) Channel patterns and terraces of the Loup River in Nebraska. US Geol Survey Prof. Paper, 422-D.https://pubs.usgs.gov/pp/0422d/report.pdf.

[45] Selby MJ (1985) Earth’s Changing Surface. Clarendon Press, Oxford.

[46] Bisson PA, Montgomery DR, Buffington JM. 2017 Valley segments, stream reaches, and channel units. In Methods in Stream Ecology, 1: 21–47. Academic Press.

[47] Old GH, Lawler DM, Snorrason A (2005) Discharge and suspended sediment dynamics of a glacial outburst flood from the Skaftá system in southern Iceland. Earth Surf Process Landf 30:1441–1460. doi: 10.1002/esp.1216

[48] Gray JR, Simões FJ (2008) Estimating sediment discharge. In: Sedimentation Engineering—Processes, Measurements,Modeling, and Practice Manual; American Society of Civil Engineers (ASCE): Reston, VA, USA, 2008; Volume 110, pp 1067–1088.

[49] Khatti J, Kaushik NP, Sharma JK, Grover KS. 2020 Modified textural soil classification. In Geotechnical Characterization and Modelling (pp. 1093–1112). Springer, Singapore.

[50] Xu ZX, Ye C, Zhang YY et al. (2020) 2D numerical analysis of the influence of near-bank vegetation patches on the bed morphological adjustment. Environ Fluid Mech 20:707–738. doi: 10.1007/s10652-019-09718-5

[51] Deb M, Das D, Uddin M (2012) Evaluation of Meandering Characteristics Using RS & GIS of Manu River. J Water Res Prot 4:163–171. doi: 10.4236/jwarp.2012.43019

[52] Bhowmik M, Das (Pan) N, Das C, Ahmed I, Debnath J (2018) Bank material characteristics and its impact on river bank erosion, West Tripura District, Tripura, North-East India. Cur Sci, 115(8):1571–1576.

[53] Debnath J, Das (Pan) N, Ahmed I, Bhowmik M (2017) Channel migration and its impact on land use/land cover using RS and GIS: a study on Khowai River of Tripura, North-East India. Egypt J Remote Sens Space Sci 20(2):197–210. doi: 10.1016/j.ejrs.2017.01.009

[54] Bevan A. (1948–1949). Floods and forestry. University of Washington Forest Club Quarterly, 22, 1–8.

[55] Sobczak D. 2015 Channel Morphology at a Meander Bend Chute Cutoff Partially Obstructed by Woody Debris.

[56] Rowntree K, Dollar ESJ (1999) Vegetation controls on channel stability in the Bell River, Eastern Cape, South Africa. Earth Surf Process Land 24(2):127–134.

[57] Jana S, Paul AK (2014) Morphodynamics of the Meandering River: A study along the Subarnarekha River of Gopiballavpur section, West Bengal, India. Int J Geol Earth Environ Sci 4(3):219–230.

[58] Inoue T, Mishra J, Kato K, Sumner T, Shimizu Y (2020) Supplied sediment tracking for bridge collapse with large-scale channel migration. Water 12(7):1881.

