# Supplementary material for: Integrated remote sensing and field-based approach to assess the temporal evolution and future projection of meanders: A case study on River Manu in North-Eastern India
Source: PLoS One. 2022 Jul 20;17(7):e0271190. doi: 10.1371/journal.pone.0271190 (PMC9299336; doi:10.1371/journal.pone.0271190)
Supplement: S2 Table — (DOCX) [file pone.0271190.s002.docx]

**Supplementary Table 2. Cross section across the Manu River at Purba Ratachhara (t2)**

| **Distance (m)** | **Reduced Level (m)** | **Water Level** |
| --- | --- | --- |
| 0 | 22.88 |  |
| 2 | 22.77 |  |
| 4 | 22.62 |  |
| 6 | 22.7 |  |
| 8 | 22.7 |  |
| 10 | 22.7 |  |
| 12 | 22.7 |  |
| 14 | 22.7 |  |
| 16 | 22.7 |  |
| 18 | 22.7 |  |
| 20 | 22.7 |  |
| 22 | 22.7 |  |
| 24 | 22.7 |  |
| 26 | 22.7 | 22.7 |
| 28 | 22.1 | 22.7 |
| 30 | 22.2 | 22.7 |
| 32 | 22.2 | 22.7 |
| 34 | 22.3 | 22.7 |
| 36 | 22.05 | 22.7 |
| 38 | 22.35 | 22.7 |
| 40 | 22.2 | 22.7 |
| 42 | 22.3 | 22.7 |
| 44 | 22.3 | 22.7 |
| 46 | 22.3 | 22.7 |
| 48 | 22.4 | 22.7 |
| 50 | 22.4 |  |
| 52 | 22.4 |  |
| 54 | 22.4 |  |
| 56 | 22.4 |  |
| 58 | 22.4 |  |
| 60 | 22.4 |  |
| 62 | 22.4 | 22.7 |
| 64 | 22.2 | 22.7 |
| 66 | 22.32 | 22.7 |
| 68 | 22.2 | 22.7 |
| 70 | 22.25 | 22.7 |
| 72 | 22.1 | 22.7 |
| 74 | 22.1 | 22.7 |
| 76 | 22.2 | 22.7 |
| 78 | 22.4 | 22.7 |
| 80 | 23 |  |
| 82 | 23.19 |  |
| 84 | 23.8 |  |
