# Supplementary material for: Integrated remote sensing and field-based approach to assess the temporal evolution and future projection of meanders: A case study on River Manu in North-Eastern India
Source: PLoS One. 2022 Jul 20;17(7):e0271190. doi: 10.1371/journal.pone.0271190 (PMC9299336; doi:10.1371/journal.pone.0271190)
Supplement: S3 Table — (DOCX) [file pone.0271190.s003.docx]

**Supplementary Table 3. Cross section across the Manu River at Fatikroy (t1)**

| **Distance (m)** | **Reduced Level (m)** | **Water Level** |
| --- | --- | --- |
| 0 | 28.3 |  |
| 2 | 27.3 |  |
| 4 | 26.9 |  |
| 6 | 26.8 |  |
| 8 | 26.7 |  |
| 10 | 25.5 |  |
| 12 | 24.35 |  |
| 14 | 24.34 |  |
| 16 | 24.33 |  |
| 18 | 24.32 |  |
| 20 | 24.31 |  |
| 22 | 24.3 |  |
| 24 | 24.29 |  |
| 26 | 24.29 |  |
| 28 | 24.29 |  |
| 30 | 24.29 |  |
| 32 | 24.285 |  |
| 34 | 24.285 |  |
| 36 | 24.283 |  |
| 38 | 24.28 |  |
| 40 | 24.28 |  |
| 42 | 24.28 |  |
| 44 | 24.28 |  |
| 46 | 24.28 |  |
| 48 | 24.28 |  |
| 50 | 24.28 |  |
| 52 | 24.26 |  |
| 54 | 24.25 | 24.25 |
| 56 | 24.25 | 24.25 |
| 58 | 24.2 | 24.25 |
| 60 | 24.1 | 24.25 |
| 62 | 24.05 | 24.25 |
| 64 | 24.1 | 24.25 |
| 66 | 23.95 | 24.25 |
| 68 | 23.7 | 24.25 |
| 70 | 23.7 | 24.25 |
| 72 | 23.6 | 24.25 |
| 74 | 23.55 | 24.25 |
| 76 | 23.45 | 24.25 |
| 78 | 23.4 | 24.25 |
| 80 | 23.4 | 24.25 |
| 82 | 23.3 | 24.25 |
| 84 | 23.2 | 24.25 |
| 86 | 23.2 | 24.25 |
| 88 | 23.25 | 24.25 |
| 90 | 23.25 | 24.25 |
| 92 | 23.1 | 24.25 |
| 94 | 23.1 | 24.25 |
| 96 | 23.1 | 24.25 |
| 98 | 23.2 | 24.25 |
| 100 | 23.2 | 24.25 |
| 102 | 23.2 | 24.25 |
| 104 | 23.2 | 24.25 |
| 106 | 23.35 | 24.25 |
| 108 | 23.4 | 24.25 |
| 110 | 23.55 | 24.25 |
| 112 | 23.7 | 24.25 |
| 114 | 23.75 | 24.25 |
| 116 | 23.9 | 24.25 |
| 118 | 24.05 | 24.25 |
| 120 | 24.15 | 24.25 |
| 122 | 24.1 | 24.25 |
| 124 | 24.15 | 24.25 |
| 126 | 24.2 | 24.25 |
| 128 | 24.23 | 24.25 |
| 130 | 24.3 | 24.25 |
| 132 | 24.35 |  |
| 134 | 24.5 |  |
| 136 | 24.7 |  |
| 138 | 24.8 |  |
| 140 | 25 |  |
| 144 | 25.2 |  |
| 146 | 25.4 |  |
| 148 | 25.6 |  |
| 150 | 25.9 |  |
| 152 | 26.3 |  |
| 154 | 26.6 |  |
| 156 | 26.8 |  |
| 158 | 27.6 |  |
| 160 | 29.3 |  |
