# Supplementary material for: Integrated remote sensing and field-based approach to assess the temporal evolution and future projection of meanders: A case study on River Manu in North-Eastern India
Source: PLoS One. 2022 Jul 20;17(7):e0271190. doi: 10.1371/journal.pone.0271190 (PMC9299336; doi:10.1371/journal.pone.0271190)
Supplement: S5 Table — (DOCX) [file pone.0271190.s005.docx]

**Supplementary Table 5. Cross section across the Manu River at Jalai (t1)**

| **Distance (m)** | **Reduced Level (m)** | **Water Level** |
| --- | --- | --- |
| 0 | 25.6 |  |
| 2 | 25.5 |  |
| 4 | 25.4 |  |
| 6 | 25.3 |  |
| 8 | 25.2 |  |
| 10 | 25.15 |  |
| 12 | 25.1 |  |
| 14 | 25 |  |
| 16 | 24.95 |  |
| 18 | 24.9 |  |
| 20 | 24.8 |  |
| 22 | 24.7 |  |
| 24 | 24.65 |  |
| 26 | 24.6 |  |
| 28 | 24.5 |  |
| 30 | 24.4 |  |
| 32 | 24.35 |  |
| 34 | 24.3 |  |
| 36 | 24.2 |  |
| 38 | 24.15 |  |
| 40 | 24.1 |  |
| 42 | 24.1 | 24 |
| 44 | 23.9 | 24 |
| 46 | 23.85 | 24 |
| 48 | 23.8 | 24 |
| 50 | 23.7 | 24 |
| 52 | 23.45 | 24 |
| 54 | 23.55 | 24 |
| 56 | 23.5 | 24 |
| 58 | 23.45 | 24 |
| 60 | 23.6 | 24 |
| 62 | 23.55 | 24 |
| 64 | 23.55 | 24 |
| 66 | 23.5 | 24 |
| 68 | 23.4 | 24 |
| 70 | 23.4 | 24 |
| 72 | 23.4 | 24 |
| 74 | 23.35 | 24 |
| 76 | 23.25 | 24 |
| 78 | 23.25 | 24 |
| 80 | 23.25 | 24 |
| 82 | 23.2 | 24 |
| 84 | 23.2 | 24 |
| 86 | 23.15 | 24 |
| 88 | 23.1 | 24 |
| 90 | 23.15 | 24 |
| 92 | 23.15 | 24 |
| 94 | 23.1 | 24 |
| 96 | 23.1 | 24 |
| 98 | 23.15 | 24 |
| 100 | 23.7 | 24 |
| 102 | 24.1 | 24 |
| 104 | 24.3 |  |
| 106 | 24.6 |  |
| 108 | 24.8 |  |
| 110 | 25.1 |  |
| 112 | 25.4 |  |
| 114 | 25.7 |  |
| 116 | 25.9 |  |
| 118 | 26.2 |  |
| 120 | 26.5 |  |
| 122 | 26.8 |  |
| 124 | 27.1 |  |
| 126 | 27.4 |  |
| 128 | 27.7 |  |
| 130 | 28 |  |
| 132 | 28.2 |  |
| 134 | 28.5 |  |
| 136 | 28.8 |  |
| 138 | 29.1 |  |
| 140 | 29.4 |  |
| 142 | 29.6 |  |
| 144 | 29.9 |  |
| 146 | 30.8 |  |
