# Supplementary material for: Integrated remote sensing and field-based approach to assess the temporal evolution and future projection of meanders: A case study on River Manu in North-Eastern India
Source: PLoS One. 2022 Jul 20;17(7):e0271190. doi: 10.1371/journal.pone.0271190 (PMC9299336; doi:10.1371/journal.pone.0271190)
Supplement: S6 Table — (DOCX) [file pone.0271190.s006.docx]

**Supplementary Table 6. Cross section across the Manu River at Jalai (t2)**

| **Distance (m)** | **Reduced Level (m)** | **Water Level** |
| --- | --- | --- |
| 0 | 26.6 |  |
| 2 | 26.5 |  |
| 4 | 26.3 |  |
| 6 | 26.2 |  |
| 8 | 26 |  |
| 10 | 25.9 |  |
| 12 | 25.87 |  |
| 14 | 25.8 |  |
| 16 | 24.7 | 23.9 |
| 18 | 23.9 | 23.9 |
| 20 | 23.7 | 23.9 |
| 22 | 23.5 | 23.9 |
| 24 | 23.5 | 23.9 |
| 26 | 23.5 | 23.9 |
| 28 | 23.4 | 23.9 |
| 30 | 23.4 | 23.9 |
| 32 | 23.5 | 23.9 |
| 34 | 23.9 | 23.9 |
| 36 | 23.9 | 23.9 |
| 38 | 23.9 | 23.9 |
| 40 | 23.8 | 23.9 |
| 42 | 23.6 | 23.9 |
| 44 | 23.5 | 23.9 |
| 46 | 23.5 | 23.9 |
| 48 | 23.5 | 23.9 |
| 50 | 23.5 | 23.9 |
| 52 | 23.5 | 23.9 |
| 54 | 23.5 | 23.9 |
| 56 | 23.6 | 23.9 |
| 58 | 23.7 | 23.9 |
| 60 | 23.7 | 23.9 |
| 62 | 23.7 | 23.9 |
| 64 | 23.6 | 23.9 |
| 66 | 23.35 | 23.9 |
| 68 | 23.2 | 23.9 |
| 70 | 23.1 | 23.9 |
| 72 | 23.9 | 23.9 |
| 74 | 24.4 |  |
| 76 | 24.9 |  |
| 78 | 25.4 |  |
| 80 | 25.9 |  |
| 82 | 26.4 |  |
| 84 | 26.8 |  |
| 86 | 27.4 |  |
| 88 | 27.8 |  |
| 90 | 28.25 |  |
| 92 | 28.7 |  |
| 94 | 29.2 |  |
| 96 | 29.6 |  |
| 98 | 30.1 |  |
| 100 | 30.6 |  |
| 102 | 31.1 |  |
| 104 | 31.6 |  |
| 106 | 32 |  |
| 108 | 32.3 |  |
