# Supplementary material for: Integrated remote sensing and field-based approach to assess the temporal evolution and future projection of meanders: A case study on River Manu in North-Eastern India
Source: PLoS One. 2022 Jul 20;17(7):e0271190. doi: 10.1371/journal.pone.0271190 (PMC9299336; doi:10.1371/journal.pone.0271190)
Supplement: S11 Table — (DOCX) [file pone.0271190.s011.docx]

**Supplementary Table 11. Grain size distribution per 100 grams in the Upper and Lower bank of the selected bends**

| Seive Size | **Purba Ratacherra** | | | | **Fatikroy** | | | | **Jalai** | | | | **Chontail** | | | | | **Srirampur** | | | | |
| --- | --- | --- | --- | --- | --- | --- | --- | --- | --- | --- | --- | --- | --- | --- | --- | --- | --- | --- | --- | --- | --- | --- |
|  | t1 | t2 | t1 | t2 | t1 | t2 | t1 | t2 | t1 | t2 | t1 | t2 | | t1 | t2 | t1 | t2 | | t1 | t2 | t1 | t2 |
|  | Upper | Lower | Upper | Lower | Upper | Lower | Upper | Lower | Upper | Lower | Upper | Lower | | Upper | Lower | Upper | Lower | | Upper | Lower | Upper | Lower |
| >2 | 0 | 0 | 0.02 | 0 | 0 | 0 | 0.03 | 0 | 0 | 0.1 | 0 | 0 | | 0 | 0 | 0.11 | 0 | | 0 | 0 | 0 | 0 |
| 1 | 11.06 | 0.43 | 0.33 | 0.7 | 1.87 | 0.26 | 2.71 | 1.55 | 1.39 | 6.59 | 1.94 | 1.04 | | 0.25 | 0.09 | 3.42 | 3.59 | | 1.16 | 0.2 | 7.5 | 2.34 |
| 0.71 | 8.28 | 1.05 | 0.52 | 1.55 | 5.53 | 1.81 | 4.76 | 3.43 | 2.68 | 9.48 | 5.7 | 1.19 | | 0.19 | 0.15 | 5.83 | 9.63 | | 2.21 | 0.56 | 10.79 | 2.6 |
| 0.25 | 23.62 | 9.44 | 5.13 | 12.31 | 29.72 | 17.66 | 20.67 | 25.05 | 47.82 | 26.08 | 68.27 | 28.14 | | 9.92 | 12.44 | 22.45 | 29.63 | | 52.01 | 7.93 | 33.06 | 10 |
| 0.125 | 24.5 | 10.38 | 52.4 | 12.83 | 19.32 | 22.6 | 22 | 18.45 | 33.18 | 20.65 | 14.13 | 32.7 | | 41.73 | 55.84 | 20.54 | 14.77 | | 7.54 | 29.56 | 9.72 | 12.03 |
| 0.06 | 27.54 | 55.41 | 37.04 | 49.83 | 29.5 | 50.26 | 41.3 | 40.9 | 13.7 | 30.56 | 9.17 | 31.49 | | 42.25 | 28.1 | 41.72 | 34.68 | | 27.95 | 51.55 | 30.12 | 57.36 |
| <0.06 | 5 | 23.29 | 4.56 | 21.78 | 14.06 | 7.41 | 8.53 | 10.62 | 1.23 | 6.54 | 0.79 | 5.44 | | 5.66 | 3.38 | 5.93 | 7.7 | | 9.13 | 10.2 | 8.81 | 15.67 |
