# Supplementary material for: Integrated remote sensing and field-based approach to assess the temporal evolution and future projection of meanders: A case study on River Manu in North-Eastern India
Source: PLoS One. 2022 Jul 20;17(7):e0271190. doi: 10.1371/journal.pone.0271190 (PMC9299336; doi:10.1371/journal.pone.0271190)
Supplement: S12 Table — (DOCX) [file pone.0271190.s012.docx]

**Supplementary Table 12. Suspended sediment concentration in water at different bend sites**

|  | **Suspended Sediment concentration (mg/l)** | |
| --- | --- | --- |
|  | **t1** | **t2** |
| **Bend 1** | 200 | 240 |
| **Bend 2** | 240 | 200 |
| **Bend 3** | 160 | 320 |
| **Bend 4** | 160 | 120 |
| **Bend 5** | 200 | 160 |
