# Supplementary material for: Integrated remote sensing and field-based approach to assess the temporal evolution and future projection of meanders: A case study on River Manu in North-Eastern India
Source: PLoS One. 2022 Jul 20;17(7):e0271190. doi: 10.1371/journal.pone.0271190 (PMC9299336; doi:10.1371/journal.pone.0271190)
Supplement: S13 Table — (DOCX) [file pone.0271190.s013.docx]

**Supplementary Table 13. Suspended sediment discharge in the selected bends**

|  | **Suspended Sediment Discharge (mt/day)** | |
| --- | --- | --- |
|  | **t1** | **t2** |
| **Bend 1** | 17.56 | 33.6 |
| **Bend 2** | 85.92 | 75.27 |
| **Bend 3** | 61.04 | 143.2 |
| **Bend 4** | 73.78 | 49.17 |
| **Bend 5** | 86.26 | 37.6 |
